# Small RNA Sequence Analysis of Adenovirus VA RNA-Derived MiRNAs Reveals an Unexpected Serotype-Specific Difference in Structure and Abundance

**DOI:** 10.1371/journal.pone.0105746

**Published:** 2014-08-21

**Authors:** Wael Kamel, Bo Segerman, Tanel Punga, Göran Akusjärvi

**Affiliations:** 1 Department of Medical Biochemistry and Microbiology, Uppsala Biomedical Center, Science for Life Laboratory Uppsala University, Uppsala, Sweden; 2 National Veterinary Institute, Uppsala, Sweden; Wuhan University, China

## Abstract

Human adenoviruses (HAds) encode for one or two highly abundant virus-associated RNAs, designated VA RNAI and VA RNAII, which fold into stable hairpin structures resembling miRNA precursors. Here we show that the terminal stem of the VA RNAs originating from Ad4, Ad5, Ad11 and Ad37, all undergo Dicer dependent processing into virus-specific miRNAs (so-called mivaRNAs). We further show that the mivaRNA duplex is subjected to a highly asymmetric RISC loading with the 3′-strand from all VA RNAs being the favored strand, except for the Ad37 VA RNAII, where the 5′-mivaRNAII strand was preferentially assembled into RISC. Although the mivaRNA seed sequences are not fully conserved between the HAds a bioinformatics prediction approach suggests that a large fraction of the VA RNAII-, but not the VA RNAI-derived mivaRNAs still are able to target the same cellular genes. Using small RNA deep sequencing we demonstrate that the Dicer processing event in the terminal stem of the VA RNAs is not unique and generates 3′-mivaRNAs with a slight variation of the position of the 5′ terminal nucleotide in the RISC loaded guide strand. Also, we show that all analyzed VA RNAs, except Ad37 VA RNAI and Ad5 VA RNAII, utilize an alternative upstream A start site in addition to the classical +1 G start site. Further, the 5′-mivaRNAs with an A start appears to be preferentially incorporated into RISC. Although the majority of mivaRNA research has been done using Ad5 as the model system our analysis demonstrates that the mivaRNAs expressed in Ad11- and Ad37-infected cells are the most abundant mivaRNAs associated with Ago2-containing RISC. Collectively, our results show an unexpected variability in Dicer processing of the VA RNAs and a serotype-specific loading of mivaRNAs into Ago2-based RISC.

## Introduction

Human adenoviruses (HAd) are non-enveloped DNA viruses with a linear double-stranded DNA genome of about 30 000–40 000 base pairs. More than 60 types of HAds have been described so far. They are classified into seven distinct subgroups A to G based on immunological, biological and biochemical characteristics [1]. In general, human adenoviruses can infect a wide range of cell types, a property making adenovirus one of the most prominent viral infectious agents in mammalian cells. HAds cause a broad spectrum of acute and chronic infections, such as respiratory tract infections and diverse ocular and gastrointestinal diseases. The infections are frequent during childhood, when they tend to be self-limiting and induce serotype-specific immunity. Similarly, the adults are prone to adenovirus infections, with reported endemic occurrence of acute respiratory disease in military trainees [2] and epidemic keratoconjunctivitis (EKC) in swimming pool associated outbreaks [3]. Viruses belonging to subgroups A and F infect mainly the gastrointestinal tract whereas viruses from subgroups B, C and E infect preferentially the respiratory tract. In contrast, subgroup D viruses, which is the largest subgroup of viruses, have a broad spectra of tropism including ocular and respiratory tract infections [4].

All HAds encode at least one virus-associated RNA (VA RNA), which is homologous to the well-characterized VA RNAI from Ad2/5 (reviewed in [5]). The majority of HAds (approx. 80%) have two VA RNA genes, designated as VA RNAI and VA RNAII. Most likely the VA RNAII gene is the result of a gene duplication of VA RNAI [6]. The VA RNAs are approximately 160 nucleotides long non-coding RNAs, transcribed by RNA polymerase III that fold into highly structured RNAs resembling precursor-microRNAs (pre-miRNAs). The Ad2/5 VA RNAI has a well-characterized essential function as a competitive substrate that binds the interferon-inducible double-stranded RNA-dependent protein kinase (PKR), thereby maintaining the translational activity in the infected cells [7], [8]. In contrast to VA RNAI, the significance of VA RNAII for the virus life cycle is still to a large extent unresolved. Whereas a mutant in VA RNAII expression grows essentially as wild type [9] a double mutant in VA RNAI and VA RNAII shows an approximately 60-fold reduction in virus growth compared to a 10-fold reduction in a VA RNAI only mutant virus [10]. Taken together these results suggest that VA RNAII can enhance virus growth although with a significantly lower efficiency compared to VA RNAI.

More recent work has also shown that both VA RNAs function as decoy RNAs targeting the RNAi/miRNA machinery by acting as competitive substrates reducing the capacity of Dicer to cleave pre-miRNA substrates and blocking pre-miRNA nuclear export [11]–[13]. Collectively, the available evidence suggests that the VA RNAs may be considered as adenovirus-encoded RNAi suppressor molecules [11]. Interestingly, VA RNAI and VA RNAII can be processed by Dicer into small RNAs, so-called mivaRNAs, that are incorporated into the RNA-induced silencing complex (RISC) [11], [14], [15]. The VA RNAI-derived small RNAs are designated mivaRNAI, whereas the VA RNAII-derived small RNAs are named mivaRNAII [15].

During a lytic adenovirus infection only a small fraction (2–5%) of the Ad5 VA RNAs are processed into mivaRNAs [12]. However, since the VA RNAs accumulate in enormous quantities during a lytic infection (>10^8^ copies/cell) [16] even this low Dicer cleavage activity will generate several million copies of the mivaRNAs per infected cell at the late stage of infection. The impact of the mivaRNAs on RISC biology is potentially significant since a large fraction of the small RNAs associated with RISC in lytic high multiplicity of infection are derived from the VA RNAs [15]. In fact, the mivaRNAs have been shown to function as classical miRNAs and can regulate host cell gene expression by targeting the complementary sequences present in a 3′-UTR [17]. However, the significance of such interactions on adenovirus growth has been brought into question since mutations in the seed sequence of both the 5′- and 3′-strand of the mivaRNAI duplex did not impair lytic virus growth, at least not in established cell lines, suggesting that the RISC associated mivaRNAs from the mivaRNAI duplex do not have target mRNA interactions that are critical to establish a lytic virus infection [18].

All HAd VA RNAs are similar in length that varies between 149–177 nucleotides. The primary nucleotide sequence of the VA RNAI and VA RNAII genes from a specific HAd differs considerably in sequence although the matching VA RNA genes between members of the same subgroup are highly conserved [6]. Based on nucleotide sequence similarity, 85 distinct VA RNAs from 47 different human adenovirus serotypes have been classified into three superfamilies. All VA RNAII species were grouped into one superfamily, whereas the VA RNAI species were subdivided into two separate superfamilies [6]. They all share a similar structural arrangement with a terminal and apical stem separated by a more complex central domain. Here we have addressed the question whether the VA RNAs expressed from different adenovirus subgroups undergo Dicer-mediated processing into mivaRNAs, and whether they are similar in sequence and abundance to the mivaRNAs expressed from the well-characterized Ad5. For these experiments we selected one prototype member of subgroup B (Ad11), C (Ad5), D (Ad37), E (Ad4) (Table S1) and characterized their mivaRNA expression profile and incorporation into RISC at late times of a lytic infection. The results show that all tested HAds express mivaRNAs, but with an unexpected variability in Dicer processing and efficiency of loading of the serotype-specific mivaRNAs into Ago2-containing RISC.

## Materials and Methods

### Cell culture and virus infection

Viruses used in this study were Ad4, Ad5, Ad11 and Ad37. Virus titers were measured as fluorescence forming units (FFU) [19] using the pan-hexon antibody (MAB8052, Millipore, 1∶500 dilution). Cells (Hela and 293-Flag-Ago2 cells) were grown in Dulbecco’s modified Eagle’s medium (DMEM, Invitrogen) supplemented with 10% fetal calf serum (FCS, Invitrogen), 1% penicillin/streptomycin (PEST) at 37°C in 7% CO_2_. For infection virus stocks were diluted to 5 FFU per cell in DMEM without serum. The medium was removed from the plate, and the diluted virus inoculum was added, followed by incubation for 1 h at 37°C, in 7% CO_2_). After the 1 h incubation the medium was removed and DMEM, supplemented with 10% FCS, 1% PEST was added and plates were returned back to the CO_2_ incubator.

### siRNA knockdown

HeLa cells were transfected with 50 nmol of siRNA using Lipofectamine 2000 (Invitrogen) according to the manufacture’s instructions. To target Dicer, a pool of two siRNAs (5′-UGCUUGAAGCAGCUCUGGAtt-3′ and 5′-UUUGUUGCGAGGGCUGAUtt-3′) was used. To detect specific effects of the Dicer siRNA a scrambled ON-TARGETplus Non-targeting siRNA Pool (Thermo Scientific) was used as a control.

### RNA extraction

Cytoplasmic RNA was prepared by lysis with IsoB-NP-40 buffer (10 mM Tris-HCl [pH 7.9], 150 mM NaCl, 1.5 mM MgCl_2_, 1% NP-40), followed by two rounds of Phenol:Chloroform:Isoamylalcohol extraction and one extraction with Chloroform:Isoamylalcohol. The RNA was precipitated with Ethanol and dissolved in H_2_O. Total RNA was extracted using the TRI Reagent (Sigma) according to the manufacture’s instructions.

### RISC immunoprecipitation and Northern blot analysis

Immunoprecipitation of the Flag/HA tagged Ago2 protein was done as described previously [20]. For Northern blot analysis, RNA was separated on a denaturing 12% polyacrylamide gel and transferred to a Hybond NX membrane (Amersham Biosciences), chemically crosslinked and hybridized as described [21]. Hybridization probes were generated by 5′-end labeling of DNA oligonucleotides with γ-^32^P-ATP by using T4 Polynucleotide Kinase (NEB). Membranes were hybridized with the probe mixtures containing an equal molar concentration of serotype-specific γ-^32^P-ATP labeled oligonucleotides (for probe nucleotide sequences see Table S2). After overnight hybridization in ULTRAhyb buffer (Ambion), the membrane was washed three times for 10 min at 42°C in 3xSSC, 0.5% SDS followed by a single wash with 1xSSC, 0.5% SDS for 15 min at 42°C. Signals were detected by exposure of membranes to a PhosphorImager screen (Fuji) followed by signal analysis with the Image One software (BioRad).

### Small RNA sequencing

The cDNA library construction and RNA-seq data was generated by the service provided by the Scilifelab Uppsala (www.scilifelab.se). Enrichment of small RNAs in the size range of 10–40 nucleotides was done by using the PureLink miRNA Isolation Kit (Invitrogen) according to the manufacturer’s protocol. RNA samples were treated with tobacco acid pyrophosphatase (TAP), which removes the γ and β phosphates from the 5′-ends of RNA and thereby generates 5′-monophosphorylated RNAs. Small RNA libraries were constructed using the SOLiD Total RNA-Seq Kit (Rev B, Life Technologies). Strand specific adapters were hybridized to the RNA before reverse transcription. The cDNA was then size-selected on a 10% TBE-Urea gel (Life Technologies) and the libraries were constructed after amplification (15 cycles). Emulsion PCR was performed using the EZ Bead System (Life Technologies) and the small RNA libraries were then sequenced on the SOLiD 5500xl system (35 bp read length, Life Technologies). Sequencing reads were mapped to the corresponding adenovirus genome (Table S1) using BLASTN (no mismatch allowed). Read start site and viral small RNA read length was mapped using customized Perl scripts (available upon request). Adenovirus mivaRNA–cellular Targets predications were performed using the miRanda software package, on default settings. The list of 3′ UTR sequences of Homo sapiens cellular genes was obtained from Biomart (www.ensembl.org).

### Total protein extraction

Cells were collected and washed once with 1xPBS. 180 µl of RIPA buffer (150 mM NaCl, 50 mM HEPES [pH 7.4], 0.5% Sodium Deoxycholate, and 0.1% SDS) supplemented with 1 U/ml of Benzonase (EMD Millipore), was added and cells suspended by vortexing and incubation for 1 h at 4°C. Cells were further disrupted by addition of 10% SDS and 1M DTT to final concentrations of 1% and 100 mM followed by a boiling step of 5 min [22].

### Western blot analysis

Protein samples were separated on a 4–20% gradient precast gel (Bio-Rad). Proteins were electro-transferred (Bio-Rad blotting chamber) onto a Immobilon-FL Western blot nitrocellulose membrane (Millipore) at 200 mA for 2–6 h, using Towbin transfer buffer (25 mM Tris, 192 mM Glycine). The membrane was blocked with Odyssey blocking buffer (LI-COR) for 1 h at 4°C and thereafter incubated with the primary antibody, diluted in the blocking buffer, overnight at 4°C. The membrane was washed 4 times for 5 min in 1xPBS followed by incubation with the fluorescent secondary antibodies (LI-COR) diluted 1∶10 000 in the blocking buffer, for 1 h at room temperature. The membrane was washed as described above and finally rinsed in 1xPBS and scanned with the Odyssey scanner (LI-COR). The following primary antibodies were used: anti-laminB (1∶4000) (Abcam), anti-Ad5 (1∶5000) (Abcam), anti-TRBP (1∶1000) (Abcam), anti-Dicer (1∶1000) (Abcam), anti-Ago2 (1∶1000) (Abnova).

### ^35^S-methionine/cysteine metabolic labeling

Prior to pulse labeling of cells, the growth medium was removed and the cells were washed once in 1xPBS and incubated in Methionine/Cysteine free DMEM (Invitrogen) (supplemented with 10% FCS, 1% PEST and 2% glutamine) for 1 h. After this starvation period the medium was removed and fresh Methionine/Cysteine free DMEM supplemented with ^35^S Protein Labeling Mix (50 µCi per 2 ml) was added and the cells were further incubated for 2h in a CO_2_ incubator (37°C, 7% CO_2_). The cells were harvested and total protein extracts were prepared as above.

## Results

### The impact of different HAds on RNAi/miRNA-pathway components

**Figure 1:**
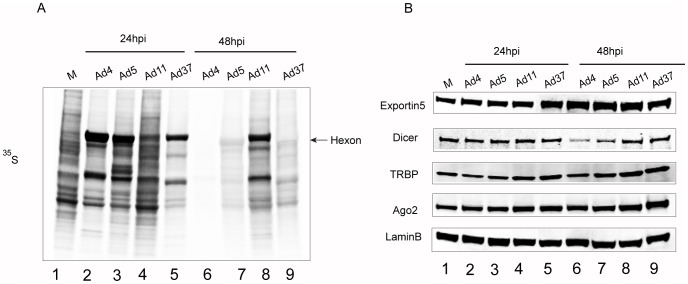
The impact of different HAd infections on RNAi/miRNA-pathway proteins. (**A**) Efficiency of different HAd infections. HeLa cells were infected with the indicated viruses, followed by a ^35^S-methionine pulse labeling after 24 and 48 hpi. Total protein lysates were separated on an SDS-PAGE and protein synthesis visualized by autoradiography. Accumulation of late viral hexon protein is indicated by an arrow. (**B**) HAd infections do not affect RNAi/miRNA-pathway protein levels. Western blot analysis on the same protein samples as in panel A was used to monitor the levels of RNAi/miRNA-pathway proteins Exportin 5, Dicer, TRBP and Ago2. Detection of the Lamin B protein served as a loading control. Letter “M” denotes mock, non-infected samples. The different panels were repeated at least two times.

Different human adenovirus serotypes show significant genome diversity, which can affect multiple aspect of the virus life cycle like cell entry, replication efficiency, interaction with the host cell pathways and the innate immune response. To monitor the lytic growth properties of HAds belonging to different subgroups we infected HeLa cells with Ad4, Ad5, Ad11 and Ad37 and followed the progress of the infection by ^35^S-methionine pulse labeling. The main read-out in this experiment is the detection of *de novo* translation of viral late proteins, which indicate the growth rate of the viruses. As shown in Figure 1A, Ad4, Ad5 and Ad37 showed an efficient production of late viral proteins, such as the hexon protein, and an efficient shut-off of host cell protein synthesis already at 24 hours post-infection (hpi) (compare lanes 2, 3 and 5 with lane 1). In contrast, the growth of Ad11 was significantly impaired and reached comparable late protein synthesis only after 48 hpi (compare lanes 4 and 8). At 48 hpi *de novo* viral protein synthesis had essentially ceased in Ad4, Ad5 and Ad37-infected cells (lanes 6, 7 and 9) indicating that the viral life cycle has reached completion for these serotypes. In conclusion, all studied serotypes actively synthesize viral late proteins at 24 hpi although Ad11 showed a slightly slower growth kinetic compared to the other three serotypes in HeLa cells.

To investigate the impact of the different HAds on the RNAi/miRNA pathway, we investigated the impact of the HAd infections on the steady state accumulation of some of the main RNAi/miRNA components (see [23] for a review); Exportin 5, Dicer, TRBP and Ago2. For this purpose HeLa cells were infected with the different HAds and the steady-state level of the proteins was monitored by Western blot analysis at 24 and 48 hpi. As shown in Figure 1B, none of the virus infections had a notable effect on the steady-state abundance of the RNAi/miRNA pathway components. The exception was the Dicer protein, which showed a slight reduction in Ad4 and possibly Ad5 infected cells at 48 hpi (lanes 6 and 7). Since the Ad4 infection was accompanied with a dramatic reduction in protein synthesis at 48 hpi (Figure 1A, lane 6) the reduction in the Dicer protein level probably reflects a turn-over of the Dicer protein in metabolically inactive cells. We conclude that HAds do not have a considerable impact on the steady-state abundance on any of the most common RNAi/miRNA pathway proteins during a short-term lytic infection (24 hpi) in HeLa cells.

### VA RNA and mivaRNA accumulation in HAd infections

**Figure 2:**
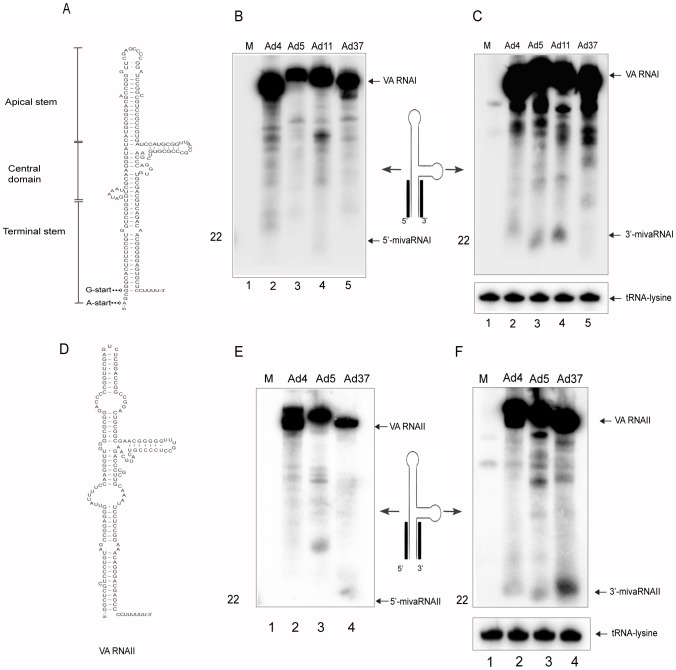
VA RNAI and VA RNAII processing in HAd infections. HeLa cells were infected with the indicated viruses. 24 hpi total RNA was extracted and analyzed by Northern blot. VA RNAI accumulation and processing into mivaRNAI was analyzed using ^32^P-labelled probes complementary to the VA RNAI 5′ stem (**B**) or the VA-RNAI 3′ stem (**C**). VA RNAII accumulation and processing into mivaRNAII was detected using ^32^P-labelled probes complementary to the VA RNAII 5′ stem (**E**) or the VA RNAII 3′ stem (**F**). The detection of tRNA-lysine served as a loading control. Horizontal black bars on the schematic VA RNAI and VA RNAII drawing indicate the localization of the Northern blot probes (Table S2). The experimentally verified secondary structures of VA RNAI (**A**) and VA RNAII (**D**) are indicated. The different panels were repeated at least two times.

The secondary structures of the VA RNAs share a great deal of similarity and fold into hairpin-like structures with a terminal and apical stem separated by a more complex central domain (for example Figure 2A, D). Previous studies have shown that the terminal stem of Ad5 VA RNAI and VA RNAII is processed into small, miRNA-like molecules by the cellular RNAi/miRNA machinery [11], [12], [14]. Due to their VA RNA descent, these small viral RNAs have been named as mivaRNAs [15]. Furthermore, depending on the exact origin of the mivaRNAs, they can be further subdivided into 5′- or 3′-mivaRNAI and 5′- or 3′-mivaRNAII (reviewed in [5]).

**Figure 3:**
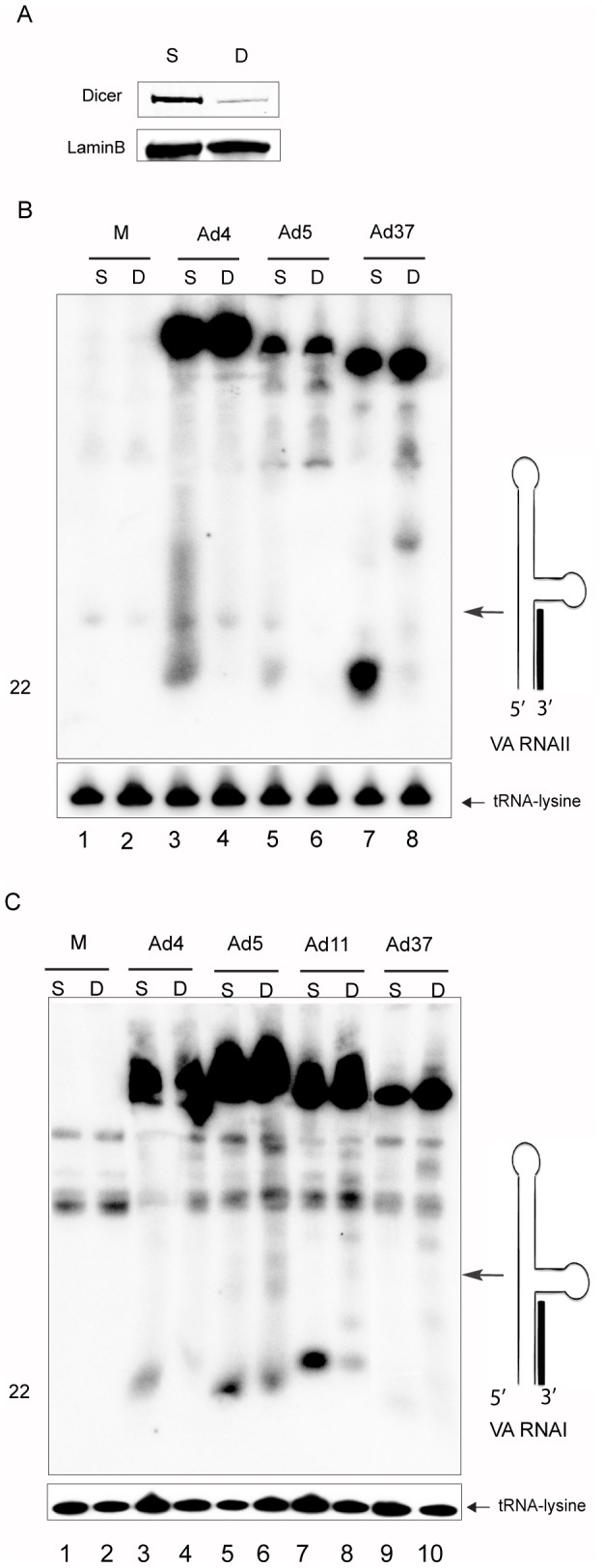
Dicer-dependent processing of the HAd mivaRNAs. (**A**) Western blot image of total Dicer protein levels in HeLa cells treated for 48 h with an siRNA directed against the Dicer protein. Panels (**B**) and (**C**), HeLa cells were transfected with a scrambled siRNA (S) or a Dicer specific siRNA (D) for 48 h, followed by infection with the indicated viruses. Total RNA was isolated 24 hpi and 3′-mivaRNAI (**B**) and 3′-mivaRNAII (**C**) accumulation analyzed by Northern blot. The detection of tRNA-lysine served as a loading control. The different panels were repeated at least two times.

Here we tested whether the VA RNAs expressed from HAds belonging to other subgroups similarly were processed into mivaRNAs. For this purpose total RNA was prepared 24 hpi from HeLa cells infected with the HAds depicted in Figure 1. The accumulation of mivaRNAs was detected by Northern blotting using ^32^P-labeled oligonucleotides complementary to the 5′- or 3′-mivaRNAI and 5′- or 3′-mivaRNAII sequences. As shown in Figure 2C Ad4, Ad5 and Ad11 expressed an easily detectable 3′-mivaRNAI (lanes 2–4). On longer exposures it became evident that also Ad37 produced low amounts of 3′-mivaRNAI with a length of approximately 22 nucleotides (data not shown). In agreement with previous results, a small RNA corresponding to the 5′-mivaRNAI accumulated in much reduced quantities in the various HAd infections (Figure 2B). The hybridization probes also detected multiple discrete longer RNA species derived from both the 5′- and 3′-end of VA RNAI (Figure 2B, C). Some of these RNA species are probably unspecific degradation products of VA RNAI or were detected due to cross-hybridization of the ^32^P-labeled oligonucleotide probes with cellular small RNAs of unknown origin (see also below, Figure 3B).

The same experimental approach was used to detect mivaRNA expression from the VA RNAII gene. Since Ad11 only contains a VA RNAI gene, the investigation was restricted to Ad4, Ad5 and Ad37. All tested serotypes expressed the 3′-mivaRNAII (Figure 2F), whereas, as expected from previous results with Ad5 [15] the 5′-mivaRNAII accumulated in much lower amounts (Figure 2E). Interestingly, Ad37 appeared to differ from the two other serotypes (Ad4, Ad5) by producing an excess of both the 5′- and 3′-mivaRNAII (see also below).

### Dicer-dependent processing of the VA RNAs

Previous results have shown that both Ad5 VA RNAI and VA RNAII are *in vitro* substrates for Dicer cleavage, generating small RNAs with a length similar to a synthetic 21-nucleotide siRNA [11], [14]. To determine whether mivaRNA processing *in vivo* also requires Dicer enzymatic activity we used an siRNA approach to knock-down the Dicer protein (Figure 3A) in HAd infected cells. For this experiment HeLa cells were pretreated with Dicer-specific or control siRNAs for 48 hours followed by infection with the indicated HAds (Figure 3B, C). Infected cells were harvested 24 hpi, total RNA was isolated and 3′-mivaRNA production visualized by Northern blot analysis. As shown in Figure 3C, knockdown of Dicer expression resulted in an essentially complete loss of 3′-mivaRNAII accumulation in all three infections (lanes 3–8) demonstrating that the VA RNAII-derived small RNAs are Dicer processing products. Similarly, knockdown of the Dicer protein resulted in a drastic reduction in VA RNAI-derived 3′-mivaRNAI accumulation (Figure 3B). However, we have consistently observed that Dicer knockdown in Ad5- and Ad11-infected cells resulted in substantial reduction but not a complete abolishment of 3′-mivaRNAI accumulation (Figure 3B, lanes 5–8). This observation is in contrast with the data from the Ad4 and Ad37 infections (Figure 3B, lanes 3, 4, 9, 10) and from 3′-mivaRNAII accumulation (Figure 3C) where we observe an almost complete loss of 3′-mivaRNA production. Interestingly, knockdown of Dicer resulted in a tiny increase in accumulation of, what appears to be, the full-length VA RNAI in Ad5, Ad11, Ad37 and VA RNAII in Ad37-infected HeLa cells and the accumulation of discrete larger degradation products that might be intermediate cleavage products (Figure 3B, C). Taken together, these results suggest that the VA RNAI and VA RNAII-derived mivaRNAs from the tested serotypes are Dicer cleavage products.

### The strand bias of mivaRNA association with RISC differs between HAds

**Figure 4:**
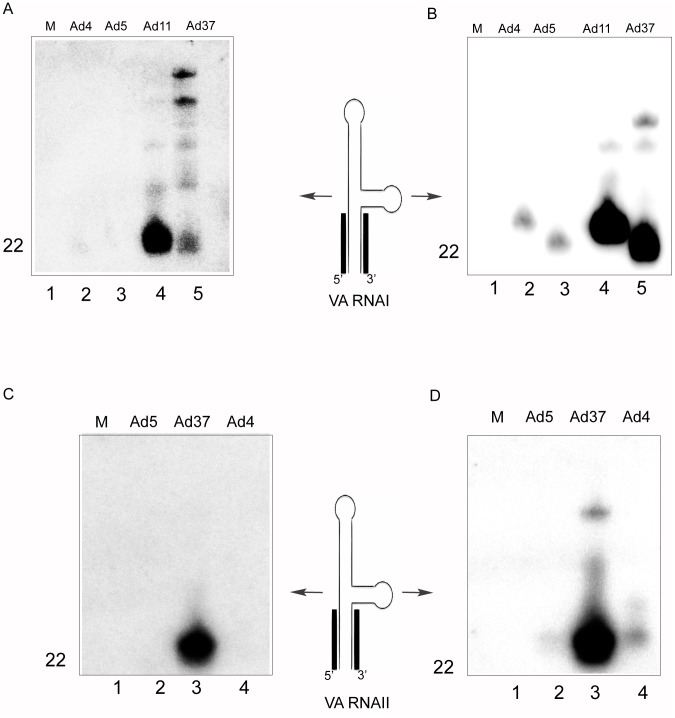
The strand bias of mivaRNA association with RISC differs between HAds. 293-Flag-Ago2 cells were infected with the indicated viruses, 24 hpi the RISC-associated small RNAs were immunopurified using an anti-FLAG resin. The Flag-Ago2 associated 5′-mivaRNAI (**A**), 3′-mivaRNAI (**B**), 5′-mivaRNAII (**C**) and 3′-mivaRNAII (**D**) were detected by Northern blot using mixtures of ^32^P-labelled oligonucleotide probes complementary to the corresponding mivaRNA sequences (Table S2). The different panels were repeated at least two times.

We have previously shown that the mivaRNAI and mivaRNAII duplexes show a highly asymmetric RISC loading in Ad5-infected cells, with the 3′-strand of both mivaRNA duplexes being >200-fold more efficiently incorporated compared to the 5′-strand [15]. To determine whether this 3′-strand bias was a conserved feature in different HAds we infected a HEK293-cell line stably expressing the FLAG/HA-epitope tagged Ago2 protein (293-Flag-Ago2; [15]), which is one of the main functional components of RISC, with the four tested HAds. Cytoplasmic S15 extracts [20] were prepared 24 hpi and FLAG/HA-Ago2 containing complexes captured by immunopurification with an anti-FLAG agarose resin. The small RNA content was probed by Northern blot analysis using ^32^P-labeled DNA oligonucleotide probes detecting either the 5′- or 3′-strand of the mivaRNAI and mivaRNAII duplexes. The successful immunopurification was indicated by the enrichment of only the processed mivaRNA and neither the full length VA RNAs nor the tRNA-lysine into RISC (Figure S1). As expected from our previous results the 3′-strand of both mivaRNAI and mivaRNAII preferentially assembled into RISC in an Ad5 infection (Figure 4B, D), although the signal was relatively weak for the 3′-strand mivaRNAII. A similar trend was observed for the 3′-mivaRNAI from Ad4 (Figure 4B). However, the most dramatic effects in mivaRNA association with Ago2 were observed for Ad11 and Ad37. Both viruses showed a dramatic increase in RISC association of mivaRNAs in 293-Flag-Ago2 cells (Figure 4). In the case of Ad11 both the 5′- and the 3′-mivaRNAI strands were well detected in RISC. However, the 3′-mivaRNAI strand bias was still retained (Figure 4A, B, see also below). Interestingly, the highly asymmetric RISC loading previously observed for Ad5 was essentially abolished for mivaRNAII in Ad37-infected cells (Figure 4C, D) where both the 5′- and the 3′-strands of the mivaRNAII duplex were loaded with a high efficiency into RISC. Taken together our data shows a serotype-specific strand bias in RISC loading of mivaRNAs.

### RNA sequence analysis of mivaRNAs in HAd-infected cells

To investigate in depth the small RNA production during different adenovirus infections, we sequenced the cytoplasmic (referred to as Cyto) and RISC bound (referred to as RISC-IP) small RNA pools isolated at 24 hpi from Ad4, Ad5, Ad11, and Ad37-infected 293-Flag-Ago2 cells. Since the VA RNAs are primary RNA polymerase III transcription products the 5′-mivaRNAs from both VA RNAs would be expected to have a mixture of mono-, di- and tri-phosphorylated 5′-ends [24]. The SOLiD sequencing technology relies on adapter ligation, which requires RNAs with a 5′ -monophosphate. To circumvent the problem that the adapter ligation would only work on a fraction of the 5′-mivaRNAs we treated the small RNA pool with TAP, which removes the γ and β phosphates from the 5′-ends of RNAs. Using this strategy we expect that the sequence information generated would more accurately represent the abundance of the 5′- and 3′-mivaRNAs.

**Figure 5:**
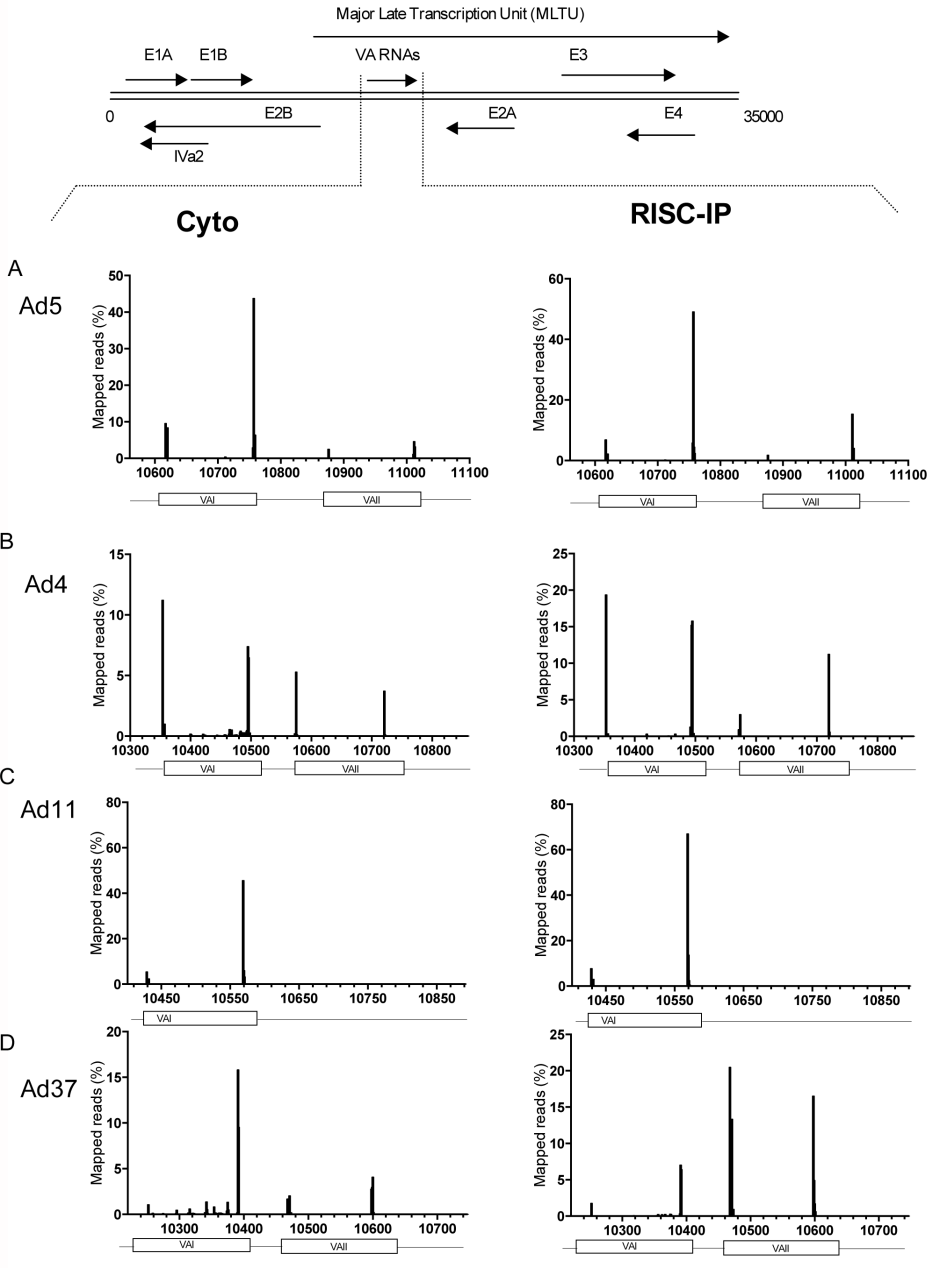
Small RNA sequencing reveals a clustering of HAd small RNAs at the VA RNAI and VA RNAII regions. The distribution profile of small RNAs expressed from the adenovirus genomes in total cytoplasmic (Cyto) and Ago2-based RISC (RISC-IP) demonstrates a mapping of small RNA sequences to the 5′ and 3′ ends of the VA RNAI and VA RNAII genomic regions (**A–D**). The numbers on the X-axis indicate the nucleotide position in respective HAd genomes. The data is shown as % of total reads aligned to the corresponding adenovirus genomes (see also Figure S3). Top, Schematic drawing showing a simplified transcription map of the Ad5 genome.

The small RNA libraries (15–35 nucleotides) prepared from cytoplasmic RNA and RISC-IP were subjected to deep sequencing using the SOLiD technology. Sequencing reads were mapped against the corresponding adenovirus genomes (Ad4, Ad5, Ad11 or Ad37). The size distribution of reads in the cytoplasmic small RNA pool showed a broad distribution of RNAs ranging from 20–35 nucleotides in length with a peak around 22 nucleotides (Figure S2). As expected the read size in the RISC-IP restricted the small RNA fraction to a sharper peak centered around 22 nucleotides, a result that indicates that the RISC immunopurification protocol enriched for small RNAs with a miRNA size. For all four serotypes, the large majority of viral small RNAs clustered around the VA region, specifically at the beginning and end of the terminal stem in VA RNAI and VA RNAII (Figure 5; Figure S3). Furthermore, the reads generated from the VA RNAs were also enriched in the RISC-IP fraction.

### Characterization of the serotype-specific mivaRNAs in the cytoplasmic and RISC bound small RNA pools

**Figure 6:**
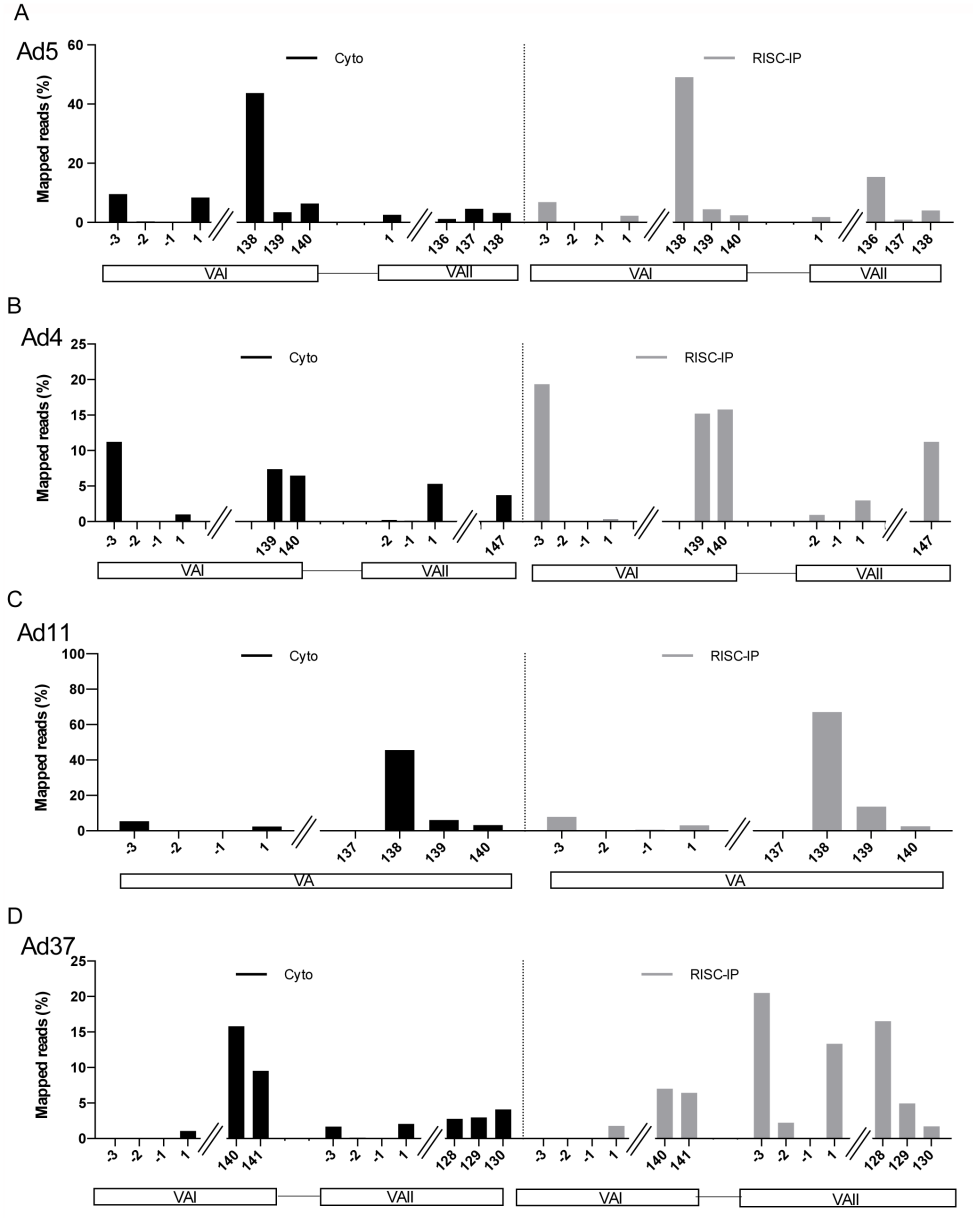
Compilation of 5′- and 3′-mivaRNA start sites. Quantitative analysis of the start position of mivaRNAI and mivaRNAII sequence reads in Ad5, Ad4, Ad11 and Ad37-infected 293-Flag-Ago2 cells. The 5′- and 3′-mivaRNAs sequences from cytoplasmic (Cyto) and RISC-associated (RISC-IP) RNA samples (Table S3) were aligned to respective serotype VA RNAI and II genome coordinates. The numbers on the X-axis indicate the exact nucleotide position of the 5′ end of the mivaRNAs expressed in the different infections with position 1 representing the classical +1 G start of the VA RNAs. The data is shown as % of total reads aligned to the corresponding adenovirus genomes.

The Ad2/5 VA RNAI gene has previously been shown to initiate transcription from two sites, generating the major G start and the minor A start located three nucleotides upstream [24], [25]. The VA RNAI(G) start accounts for approximately 75% of the total VA RNAI population in a HeLa cell infection [24]. Interestingly, the Ad4, Ad5 and Ad11 VA RNAI genes and the Ad4 and Ad37 VA RNAII genes (Figure 6) produces 5′-mivaRNAs corresponding to transcription initiation events at an upstream A start in addition to the conventional +1 G start (see also Table S3). Further, the Ad4 VA RNAII(A) start differs from the others by initiating two nucleotides upstream of the G start site instead at the typical −3 position (Figure 6). The Ad37 VA RNAII differs even further by showing evidence of the usage of three alternative start sites for transcription: an A start (−3), a G start (−2) and the conventional G start at +1 (Figure 6). It is noteworthy that the mivaRNAs with the upstream start sites (the A start) are in all cases preferentially incorporated into RISC. The most extreme example is the Ad4 VA RNAI, where the RISC associated 5′-mivaRNAI(A) accounts for more than 95% of 5′-strands in RISC (Figure 6; Table S3).

Previous studies have shown that the Ad5 3′-mivaRNAI and 3′-mivaRNAII are heterogeneous at their 5′-terminus suggesting that Dicer cleavage in the terminal stem varies slightly (see Discussion). Also, since the length of the VA RNAs varies slightly (Table S3) the numbering differs somewhat between serotypes. As shown in Figure 6, approximately 90% of all 3′-mivaRNAs in both the cytoplasmic and RISC-IP in Ad5 and Ad11 VA RNAI originated from a single cleavage event generating Ad5 and Ad11 3′-mivaRNAI-138. In contrast, in Ad4 and Ad37-infected cells two alternative cleavage events appears to generate two 3′-mivaRNAI species which accumulates in approximately equal quantities in the RISC-IP fraction (Ad4 3′-mivaRNAI-139 and −140; Ad37 3v-mivaRNAI-140 and −141).

The VA RNAII genes differ significantly in length between the three serotypes tested (Table S1). Therefore, the numbering of the 3′-mivaRNAII species differs significantly although Dicer cleavage occurs at the same region in the terminal stem. Whereas the 3′-mivaRNAII-147 was, by far, the most dominant Ad4 3′-mivaRNAII in RISC-IP both Ad5 and Ad37 showed a larger heterogeneity in 3′-mivRNAII abundance in RISC (Figure 6; Table S3). Thus, in Ad37 3′-mivaRNAII-128 was most abundant in RISC-IP (Figure 6; 17% of total reads) with a gradual decrease in cut site generating 3′-mivaRNAII-129 to −131. Interestingly, the reads corresponding to Ad37 3′-mivaRNAII-128 to −130 were approximately equal in the cytoplasmic pool whereas the 3′-mivaRNAII-128 was the preferred small RNA detected in the RISC-IP (Figure 6). Similarly, Ad5 3′-mivaRNAII-137 was the most abundant small RNA in the cytoplasmic fraction whereas 3′-mivaRNAII-136 and 3′-mivaRNAII-138 surpassed it in the RISC-IP (Figure 6).

Collectively our results suggest that the asymmetry in RISC loading observed in Ad5-infected cells [15] extends to the HAds analyzed here. Thus, the 3′-mivaRNAs derived from the VA RNAI and VA RNAII transcripts in all serotypes were the preferred strands for RISC loading, except for the Ad37 VA RNAII, where the 5′-mivaRNAII was the favored guide strand found in RISC (Figure 6; Table S3).

## Discussion

Here we have characterized mivaRNA expression from four HAds belonging to different subgroups (Table S1) in order to establish similarities and differences in mivaRNA structure and expression. The data show that the VA RNAs from all tested serotypes are processed by the Dicer enzyme (Figure 3) into small RNAs that resemble in size mature cellular miRNAs. Further we show that the large majority of all small RNAs derived from the adenovirus genome are clustered around the VA RNA region (Figures S4 and S5). Also, these VA RNAI and VA RNAII-derived mivaRNAs are significantly enriched in the RISC complex (Figures 4–6). These observations indicate that the mivaRNAs expressed from different HAds potentially may function as classical miRNAs regulating cellular and/or viral targets. Evidence has been presented indicating that mivaRNAs expressed at different time points of infection might serve specific functions [26]. Although attractive this conclusion has recently been challenged since Ad5 mutant viruses with mutations disrupting the mivaRNAI 5′- or 3′-seed sequences replicate as the wild type virus, at least in some tested human cell lines [18]. This finding questions the significance of any potential mivaRNA interaction with cellular and/or viral target RNAs during a short-term lytic Ad5 infection. However, it should be noted that this finding does not exclude the possibility that mivaRNA regulation of cellular and/or viral target RNA expression plays an important role for HAd multiplication in its natural host or during the establishment of long term persistent infections.

**Figure 7:**
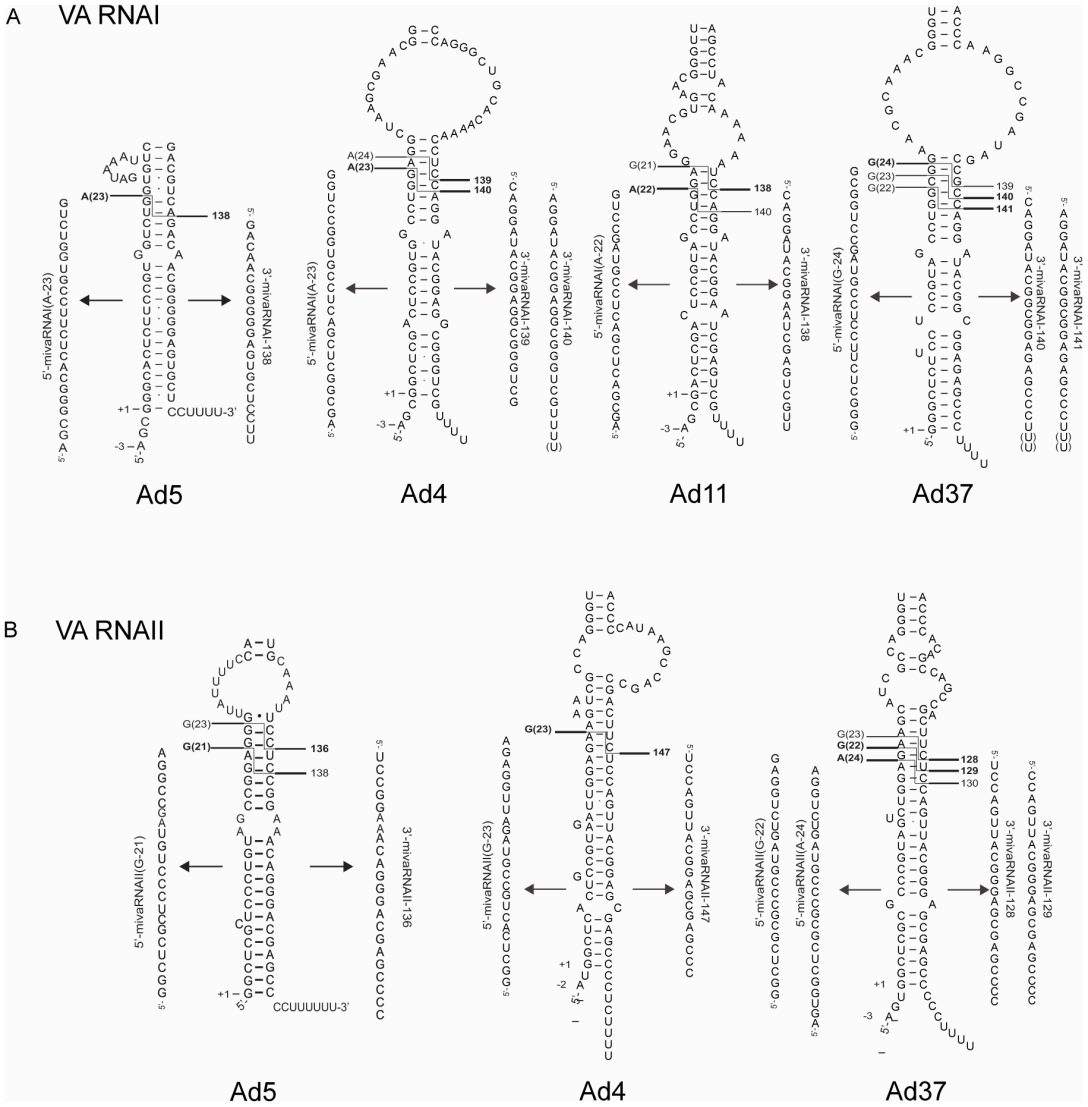
Deduced Dicer cleavage sites on the VA RNAs. Schematic drawing illustrating the predicted Dicer cleavage sites needed to generate the major RISC-associated 5′- and 3′-mivaRNAs expressed from the VA RNAI (**A**) and VA RNAII (**B**) transcripts, respectively. In some VA RNAs two mivaRNA are shown because they are expressed at similar levels (Table S3).

In mammalian cells Dicer recognizes stem-loop structured pre-miRNAs and cleaves both strands to generate 21–25 nucleotide dsRNA duplexes with a two nucleotide 3′-overhang and a phosphate group at the recessed 5′-end (reviewed in [27]). The exact Dicer processing site is important since variations in cleavage site selection generates miRNAs with different seed sequences [28] It is noteworthy that the actual Dicer cleavage event in the terminal stem of the VA RNAs from the different serotypes is not unique, as illustrated by the production of 5′-mivaRNAs with a varying length and 3′-mivaRNAs with a slight variation of the position of the 5′-nucleotide (Table S3). Figure 7 summarizes the sequence(s) of the most abundant RISC associated 5′- and 3′-mivaRNAs accumulating in the different HAd infections. The results suggest that the alternative Dicer cleavage events contribute to the abundance of different 5′- and 3′-mivaRNAs in the infected cells. For example, in Ad5 VA RNAII the major 21 nucleotide long 5′-mivaRNAII(G-21) is derived from the Dicer product that also generates the minor 3′-mivaRNAII-138 whereas the minor 23 nucleotide long 5′-mivaRNAII(G-23) is derived from the Dicer product that generated the major 3′-mivaRNAII-136 guide RNA (Figure 7B; Table S3). In contrast, one Dicer cleavage event in Ad5 VA RNAI appears to be sufficient to generate both the major 5′-mivaRNAI(A-23) and 3′-mivaRNAI-138 (Figure 7A; Table S3). Based on the sequence read count the 3′-strand of this dsRNA duplex appears to function as guide strand with an approximately 6.5-fold higher efficiency (Table S3).

**Figure 8:**
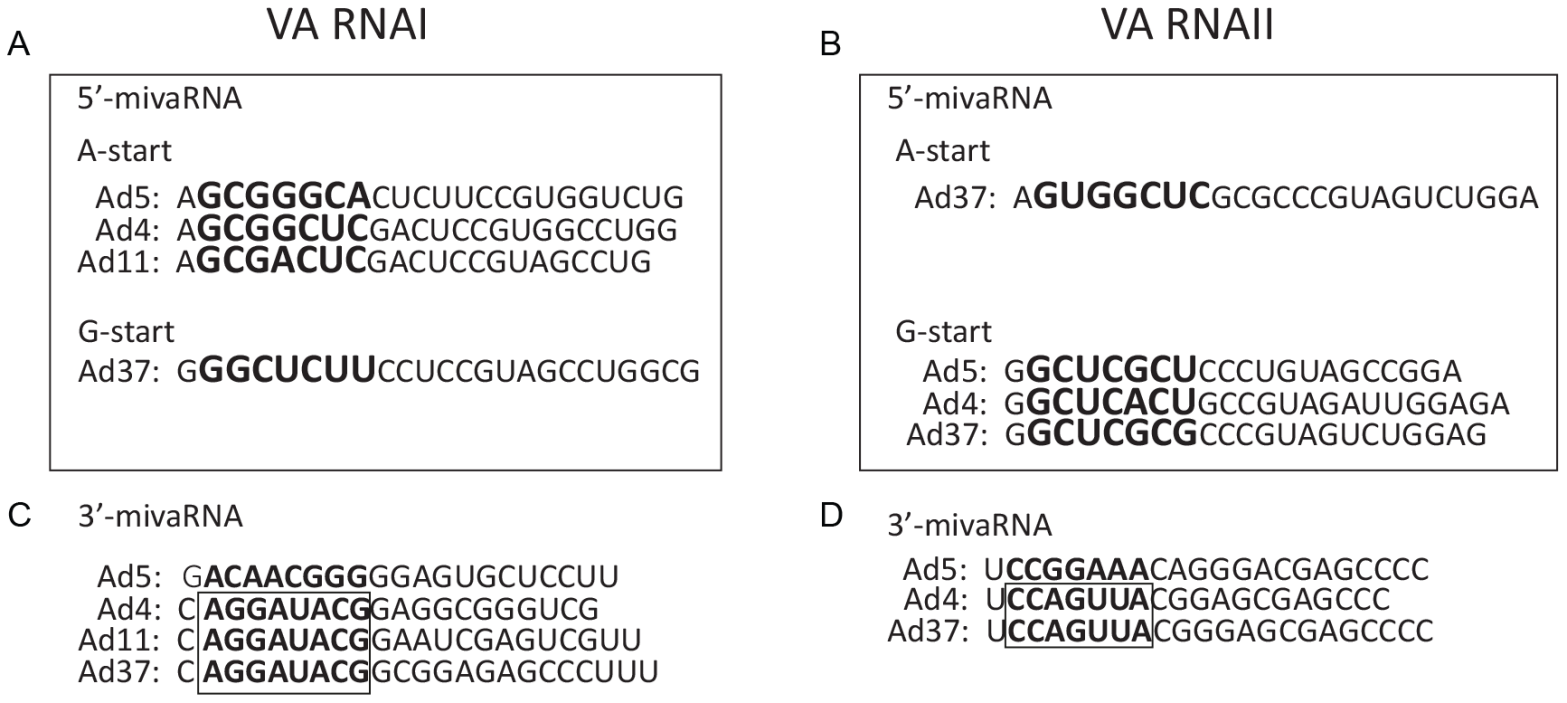
Alignment of mivaRNA seed sequences. The sequence of the major 5′-mivaRNAI (**A**) and 5′-mivaRNAII (**B**) are grouped into A-start and G-start mivaRNAs. The sequence of the 3′-mivaRNAI (**C**) and 3′-mivaRNAII (**D**) are shown with the seed sequence for the most abundant 3′-mivaRNAs shown in bold.

Interestingly, our analysis also suggests that the utilization of two alternative start sites in VA RNA transcription appears to be the rule rather than an exception. Thus, all analyzed VA RNAs, with the exception of Ad37 VA RNAI and Ad5 VA RNAII, appears to have an alternative upstream start site to the classical +1 G start (Figures 6–8; Table S3). A closer examination of the mivaRNA production in these serotypes further support the observation that alternative Dicer cleavage positions in the terminal stem of the VA RNAs generate the 5′- and 3′-mivaRNAs that function as guide strands in RISC assembly (Figure 7).

A recent study has established that the precise Dicer cleavage position can be controlled by accurate loop positioning in the stem-loop structured pre-miRNA [29] This, so-called “loop-counting rule” implies that Dicer cuts dsRNA precisely after detection of a single-stranded RNA sequence, either from the loop region or internal bulge, at a fixed 2-nucleotide distance relative to the site of cleavage. We applied the “loop-counting rule” to our studied VA RNA structures and found that a single major Dicer cleavage event takes place in Ad5 VA RNAI (Figure 7A) and Ad4 VA RNAII (Figure 7B), producing the major dsRNA duplex used in RISC assembly.

Structural studies and bioinformatics analyses have shown a clear preference for a 5′-U in miRNA guide strands assembled into functional RISC [30], [31]. Intriguingly, our deep sequencing analysis experimentally confirms that mivaRNA strands beginning with a 5′-U are favored in loading into Ago2-containing RISC. This 5′-U bias in RISC loading is a common property shared between Ad4 mivaRNAII-147, Ad5 mivaRNAII-136 and Ad37 mivaRNAII-128. These 3′-mivaRNAIIs were enriched from 3.7%, 1% and 2.7% in the cytoplasmic fraction to 11.2%, 15.3% and 16.5% in the RISC fraction, respectively (Figure 6; Table S3). Interestingly, during a long term persistent Ad5-infection in lymphoid cells, mivaRNAII-136 was also the dominant mivaRNA found in the RISC fraction [32].

To better understand the potential function of serotype-specific mivaRNAs we examined the seed sequence conservation [33] among our newly characterized mivaRNAs. Previous experiments have suggested that in Ad5 the 5′-mivaRNAI processed from VA RNAI(A-23) is more efficiently assembled into RISC and generates active RISC complexes, whereas the 5′-mivaRNAI generated from VA RNAI(G-21) is inefficient in RISC loading and essentially nonfunctional in target RNA cleavage [25]. Further, our experiments suggested that the Ad5 3′-mivaRNAI-138 was the preferred substrate in RISC loading but generated RISC complexes with an *in vitro* cleavage activity of only 2% compared to the 5′-mivaRNAI(A) strand. From this point it is interesting to note that in three of the analyzed HAds the 5′-mivaRNAI generated from the VA RNAI A start is the predominant 5′-mivaRNAI in RISC (Figure 6). Further, the seed sequences are well conserved between Ad4 and Ad11 (one mismatch) and show a significant homology also to the Ad5 5′-mivaRNAI(A) (Figure 8A). Mismatches in the seed interaction can sometimes be compensated by additional pairing interactions centered around nucleotides 13–17 in the miRNA (reviewed in [33]). Ad37 does not produce a detectable 5′-mivaRNAI(A) species from VA RNAI, but instead produces large amounts of RISC associated A and G start 5′-mivaRNAs from its VA RNAII gene. It is noteworthy that the seed sequence of the Ad37 5′-mivaRNAII(A) strand is homologous to the Ad4 5′-mivaRNAI(A) seed sequence (one mismatch; Figure 8). Moreover, in Ad5, Ad4 and Ad37 where the 5′-mivaRNAII(G) strand is preferentially associated with RISC the seed sequences show a high degree of homology (Figure 8B).

In all our studied HAds there is a clear enrichment of 3′-mivaRNAs in RISC-IP samples (Figures 4 and 6). As shown in Figure 8C the Ad4, Ad11 and Ad37 3′-mivaRNAI have a conserved seed sequence. All three serotypes belong to VA RNA superfamily 2 [6]. In contrast Ad5, which belongs to VA RNA superfamily 1 has 3 mismatches in the seed sequence. Also, the Ad4 3′-mivaRNAII-147 and Ad37 3′-mivaRNAII-128 have an identical seed sequence whereas the Ad5 3′-mivaRNAII-136 seed sequence differs at 3 nucleotide positions compared to Ad4 and Ad37 seed sequence (Figure 8D). Based on this simple seed sequence comparison we would expect that the Ad4, Ad11, Ad37 3′-mivaRNAs might target cellular and/or viral mRNAs, which would not be addressed by the Ad5 3′-mivaRNAs. As we show here the 5′-end of the 3′-mivaRNAs from both VA RNAI and VA RNAII are heterogeneous in all four HAds (Table S3), most likely due to variations in Dicer processing (see above), 3′-mivaRNAs with altered seed sequences could generate viral miRNAs with different targeting properties. A bioinformatic analysis, based on miRBase database search, indicates that the 3′-mivaRNAs described in this study do not share a seed sequence with any cellular or viral miRNA so far characterized.

**Figure 9:**
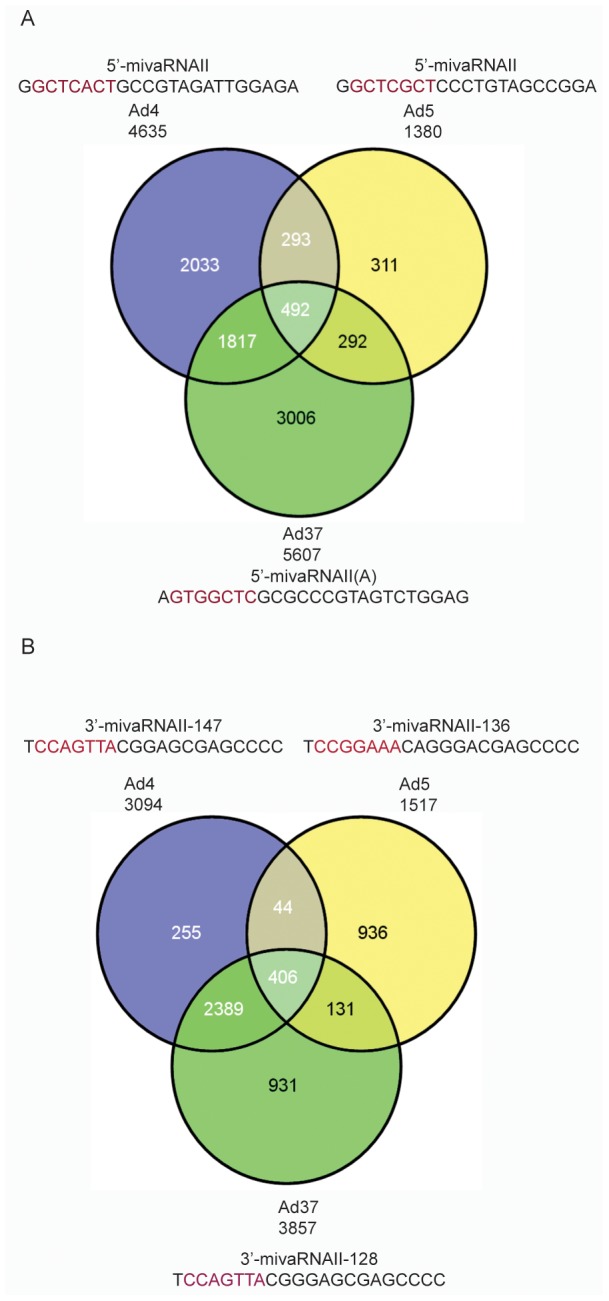
Computational predication of mivaRNAII target genes. The number of targeted human genes by the most abundant small RNA generated from the Ad4, Ad5 and Ad37 5′- (**A**) or 3′- (**B**) mivaRNAII were predicated using the miRanda software and presented as a Venn diagram. Numbers in the intersecting circles indicate the number of genes predicated to be common targets by the mivaRNAIIs from the different HAds.

In this study we investigated the potential function of the adenovirus-encoded mivaRNAs by an alternative, evolutionary approach. Although our data indicates that the seed sequences vary slightly between the mivaRNAs they may still target the same cellular genes by having alternative 3′ UTR interactions. To test the target conservation the major RISC associated mivaRNAs from the different HAds were analyzed using the miRanda software package [34]. The conservation is most impressive for the mivaRNAs derived from the VA RNAII transcripts. Thus, 78% of the Ad5 5′-mivaRNAII cellular targets were shared with at least one other serotype and of these almost 36% were shared between all three serotypes (Figure 9A). Similarly, 27% of the 3′-mivaRNAII targets were conserved between the three serotypes (Figure 9B). The VA RNAI derived mivaRNAs showed a significantly lower degree of conservation: an approximately 2% conservation of cellular targets for the Ad5 5′- and 3′-mivaRNAIs, respectively (Figure S5). Future work will focus on experimentally validating the conserved targets, and investigations on the significance of these for the adenovirus life cycle.

## Supporting Information

Figure S1 **Incorporation of the processed mivaRNA I and II into RISC.** 293-Flag-Ago2 cells were infected with the indicated viruses, 24 hpi the RISC-associated small RNAs were immunopurified using an anti-FLAG resin. 5′-mivaRNAI (**A**), 3′-mivaRNAI (**B**), 5′-mivaRNAII (**C**) and 3′-mivaRNAII (**D**) were detected both in the input and RISC fractions by Northern blot using mixtures of ^32^P-labelled oligonucleotide probes complementary to the corresponding mivaRNA sequences (Table S2). The detection of tRNA-lysine served as a loading control.
(TIF)

Figure S2 **Size distribution of the viral small RNA reads in the cytoplasmic (Cyto) and RISC-bound (RISC-IP) fraction in 293-Ago2-infected cells.**
(TIF)

Figure S3 **Mapping of Ad4 and Ad5 small RNA reads to the 5**′**- and 3**′**-end of the VA RNA genes.** The data shown is similar to Fig. 5 but here presented as number of reads.
(TIF)

Figure S4 **Mapping of Ad11 and Ad37 small RNA reads to the 5**′**- and 3**′**-end of the VA RNA genes.** The data shown is similar to Fig. 5 but here presented as number of reads.
(TIF)

Figure S5 **Computational prediction of mivaRNAI target genes.** The number of targeted human genes by the most abundant small RNA generated from the Ad4, Ad5, Ad11 and Ad37 5′- (A) or 3′- (B) mivaRNAI were predicted using the miRanda software and presented as a Venn diagram. Numbers in the intersecting circles indicate the number of genes predicted to be a common target by the mivaRNAIs from the different HAds.
(TIF)

Table S1 **List of the human adenovirus (HAd) serotypes used in this study.**
(PDF)

Table S2 **Nucleotide sequences of DNA oligonucleotides used.**
(PDF)

Table S3 **List of total reads and length variations of the mivaRNAs expressed in the HAd infections.**
(PDF)
