# Supplementary figures and images for: Small RNA Sequence Analysis of Adenovirus VA RNA-Derived MiRNAs Reveals an Unexpected Serotype-Specific Difference in Structure and Abundance
Source: PLoS One. 2014 Aug 21;9(8):e105746. doi: 10.1371/journal.pone.0105746 (PMC4140831; doi:10.1371/journal.pone.0105746)

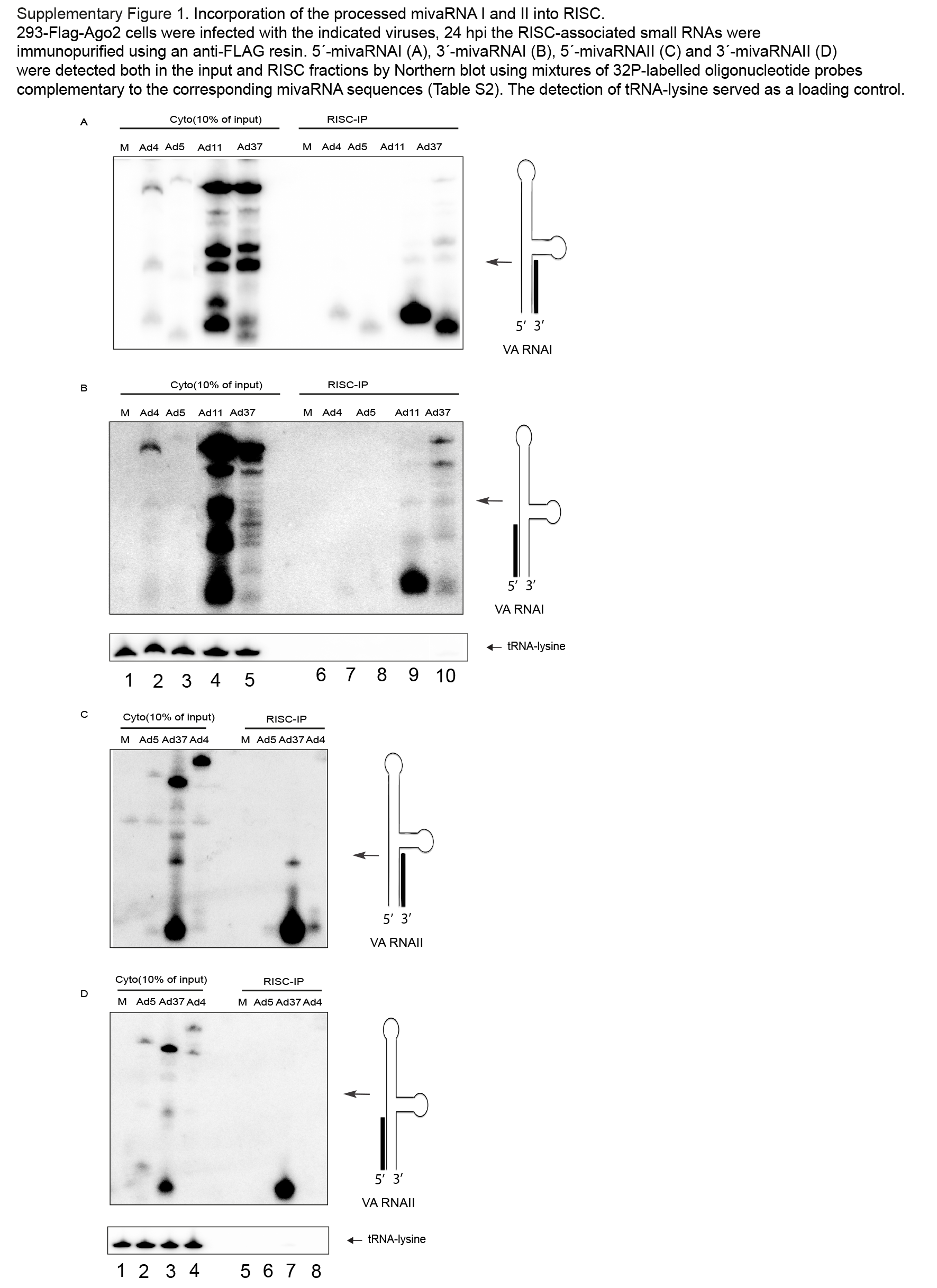

Supplement: Figure S1 — Incorporation of the processed mivaRNA I and II into RISC. 293-Flag-Ago2 cells were infected with the indicated viruses, 24 hpi the RISC-associated small RNAs were immunopurified using an anti-FLAG resin. 5′-mivaRNAI (A), 3′-mivaRNAI (B), 5′-mivaRNAII (C) and 3′-mivaRNAII (D) were detected both in the input and RISC fractions by Northern blot using mixtures of 32P-labelled oligonucleotide probes complementary to the corresponding mivaRNA sequences (Table S2). The detection of tRNA-lysine served as a loading control. (TIF) [file pone.0105746.s001.tif]

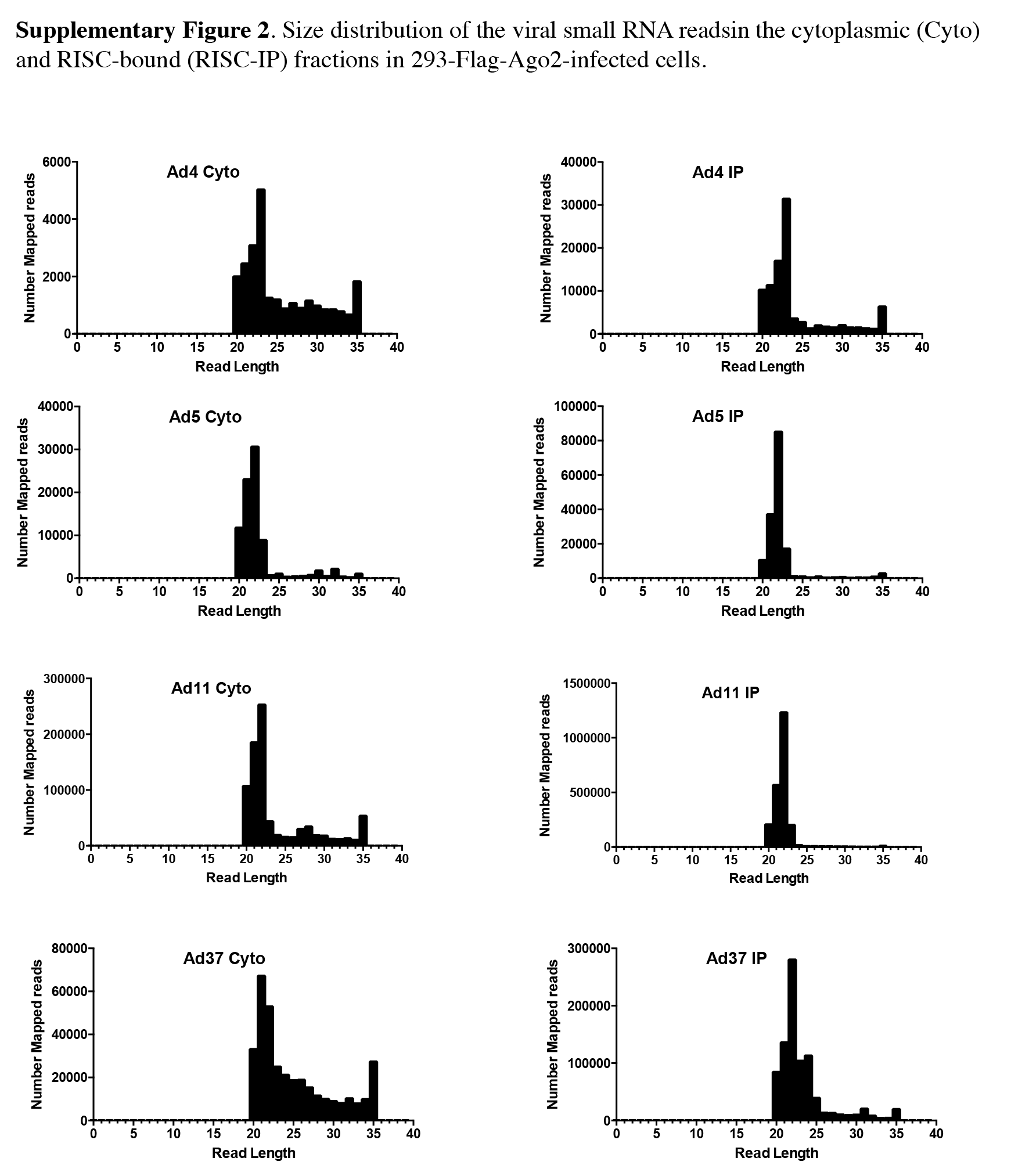

Supplement: Figure S2 — Size distribution of the viral small RNA reads in the cytoplasmic (Cyto) and RISC-bound (RISC-IP) fraction in 293-Ago2-infected cells. (TIF) [file pone.0105746.s002.tif]

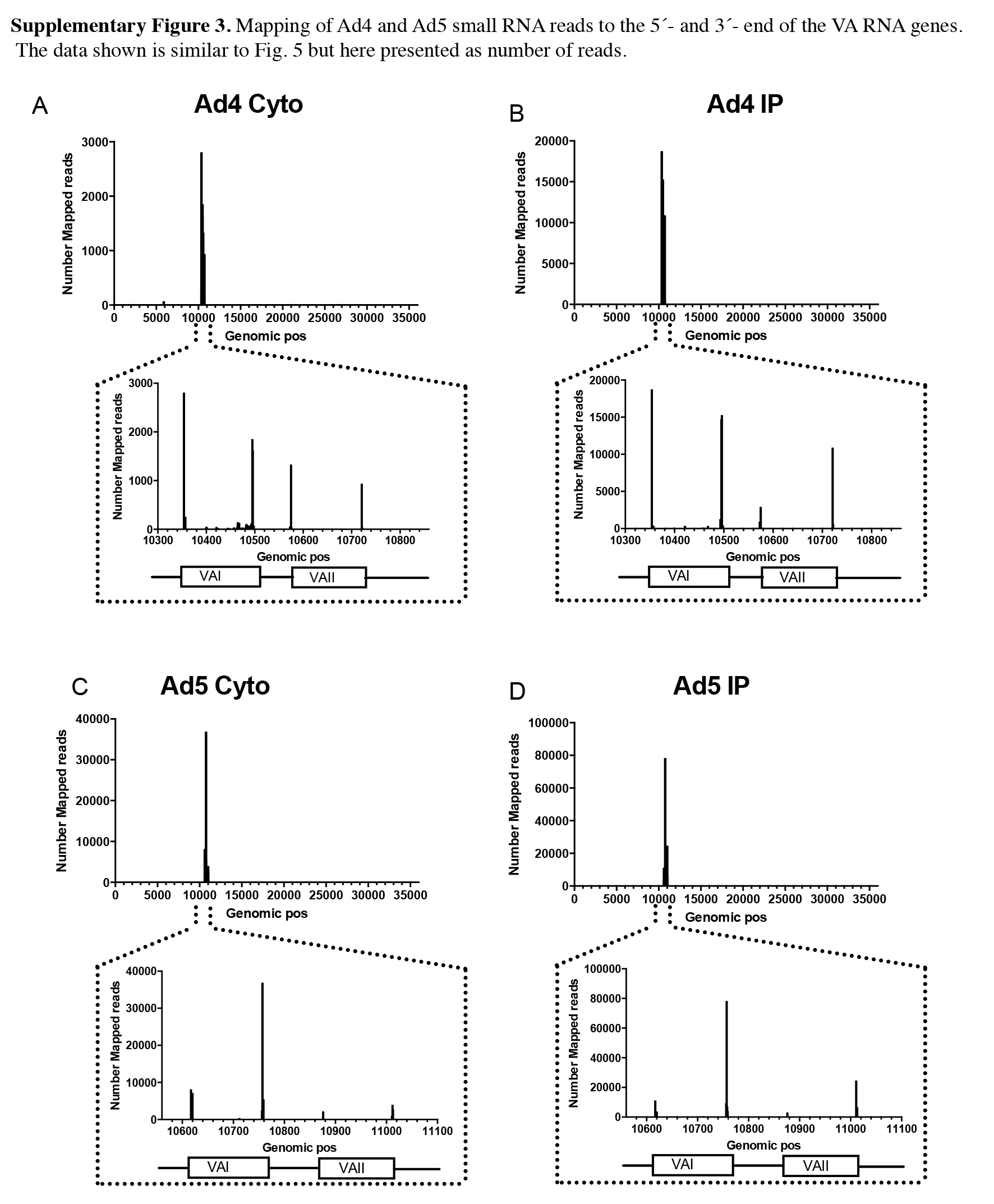

Supplement: Figure S3 — Mapping of Ad4 and Ad5 small RNA reads to the 5′- and 3′-end of the VA RNA genes. The data shown is similar to Fig. 5 but here presented as number of reads. (TIF) [file pone.0105746.s003.tif]

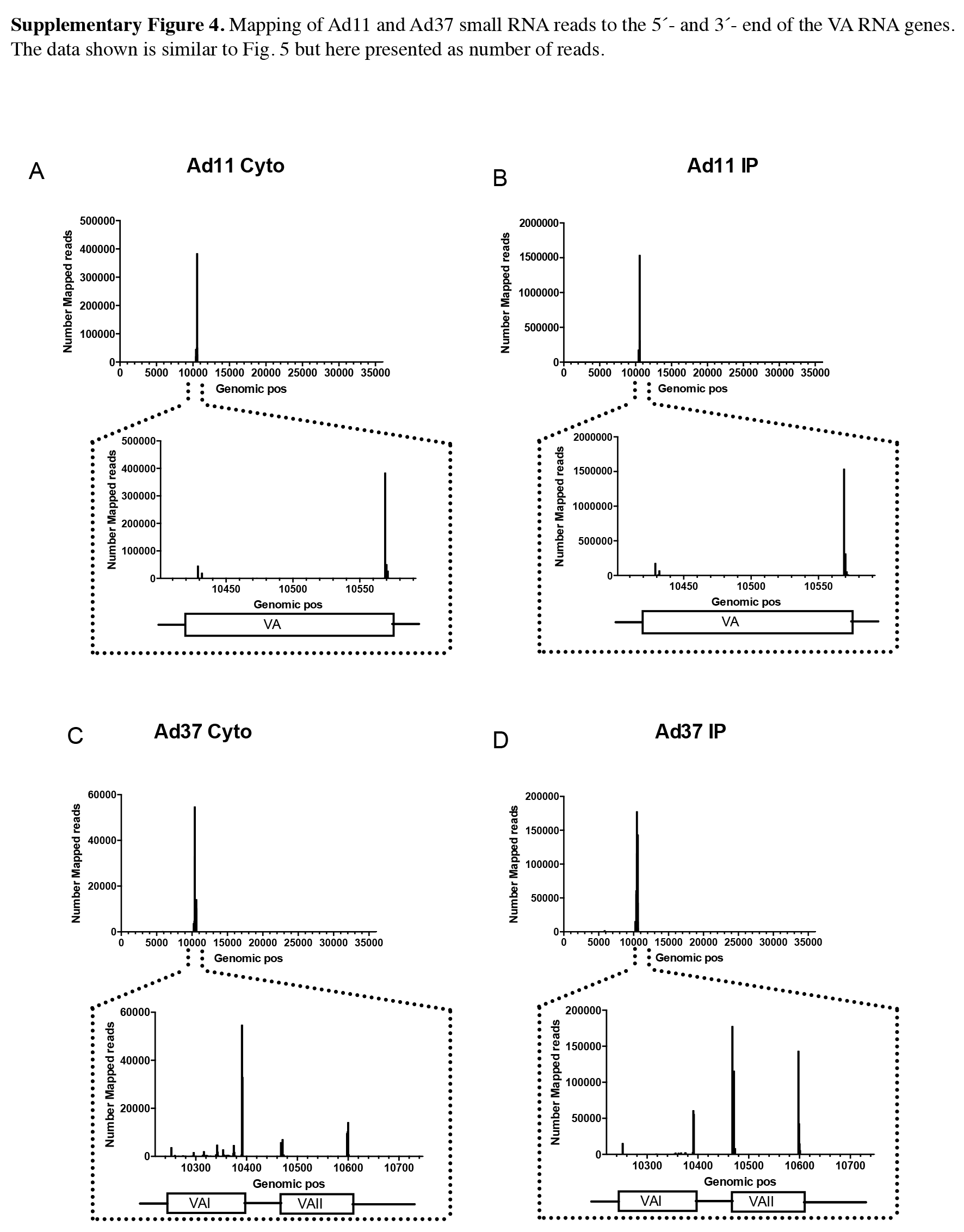

Supplement: Figure S4 — Mapping of Ad11 and Ad37 small RNA reads to the 5′- and 3′-end of the VA RNA genes. The data shown is similar to Fig. 5 but here presented as number of reads. (TIF) [file pone.0105746.s004.tif]

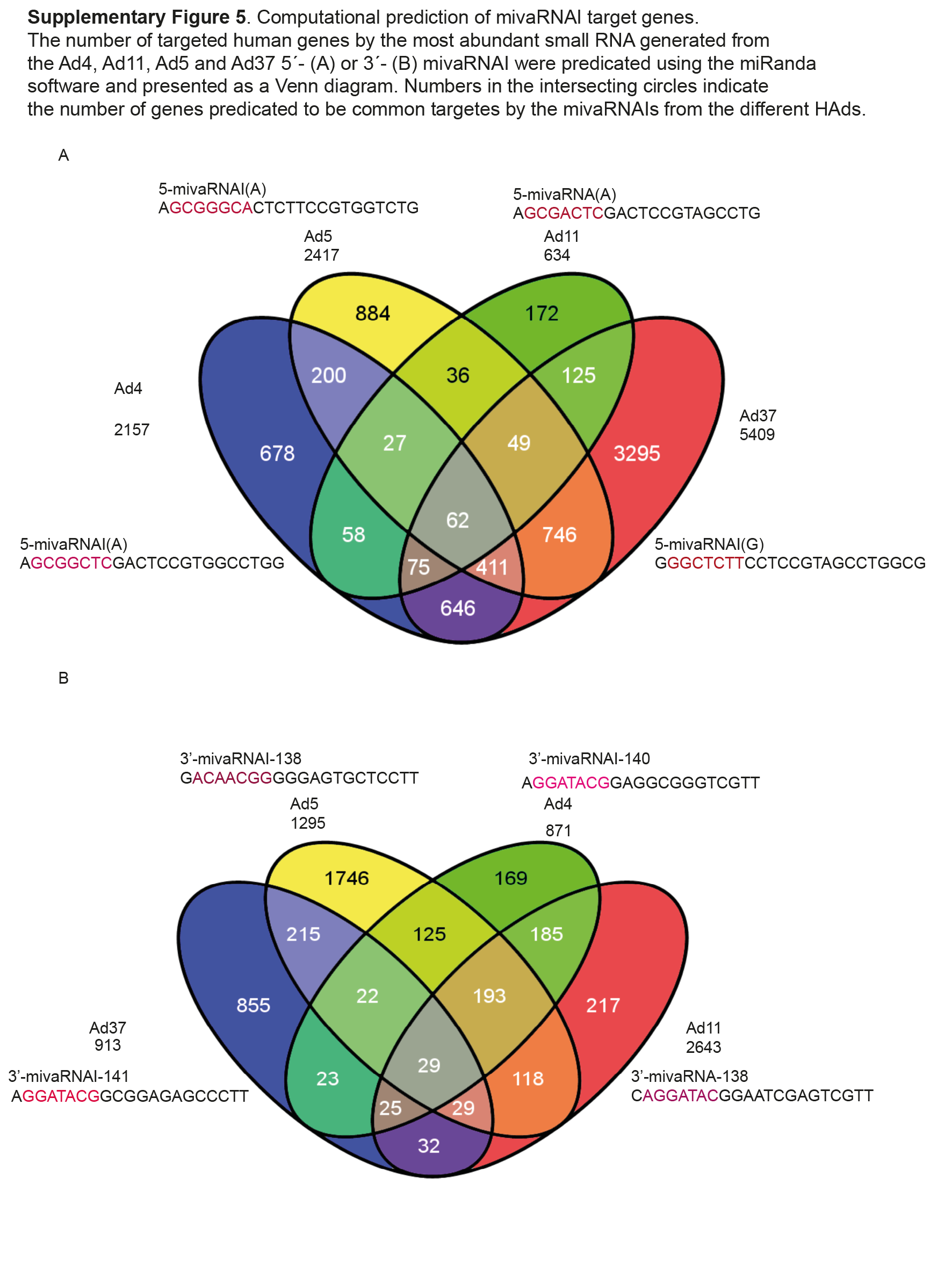

Supplement: Figure S5 — Computational prediction of mivaRNAI target genes. The number of targeted human genes by the most abundant small RNA generated from the Ad4, Ad5, Ad11 and Ad37 5′- (A) or 3′- (B) mivaRNAI were predicted using the miRanda software and presented as a Venn diagram. Numbers in the intersecting circles indicate the number of genes predicted to be a common target by the mivaRNAIs from the different HAds. (TIF) [file pone.0105746.s005.tif]
