# Supplementary material for: Small RNA Sequence Analysis of Adenovirus VA RNA-Derived MiRNAs Reveals an Unexpected Serotype-Specific Difference in Structure and Abundance
Source: PLoS One. 2014 Aug 21;9(8):e105746. doi: 10.1371/journal.pone.0105746 (PMC4140831; doi:10.1371/journal.pone.0105746)
Supplement: Table S1 — List of the human adenovirus (HAd) serotypes used in this study. (PDF) [file pone.0105746.s006.pdf]

Supplementary Table 1. List of the human adenovirus (HAd) serotypes used in this study.

Serotype	Subgroup	Genome size (BP)	VA gene(s)	VA genes length(nt)	NCBI accession no.	Receptor
Ad5	C	35938	VAI	160	AC_000008.1	CAR, integrin αvβ
			VAII	161		
Ad4	E	35994	VAI	162	NC_003266	CAR, integrin αvβ
			VAII	173		
Ad11	B2	34794	VA	161	AY163756	CD46, integrin αvβ
Ad37	D	35213	VAI	163	DQ900900	CD46, sialic acid, integrin αvβ
			VAII	150		
