# Supplementary material for: Small RNA Sequence Analysis of Adenovirus VA RNA-Derived MiRNAs Reveals an Unexpected Serotype-Specific Difference in Structure and Abundance
Source: PLoS One. 2014 Aug 21;9(8):e105746. doi: 10.1371/journal.pone.0105746 (PMC4140831; doi:10.1371/journal.pone.0105746)
Supplement: Table S2 — Nucleotide sequences of DNA oligonucleotides used. (PDF) [file pone.0105746.s007.pdf]

Supplementary Table 2. Nucleotide sequences of DNA oligonucleotides used.

Name	Sequence 5' to3'
**3'end Ad5-VARNAI**	AAAAGGAGCACTCCCCCGTTGTCTGACGTCGCA
**3'end Ad4-VARNAI**	AAAACGACCCGCCTCCGTATCCTGGAGGTTTTG
**3'end Ad11-VARNAI**	AAAACGACTCGATTCCGTATCCTGGATTTTTGT
**3'end Ad37- VARNAI**	TTTGCGTTCCGCCAGGCTACGGAGGAAGAG CCC
**5'end Ad5-VARNAI**	GAATTTATCCACCAGACCACGGAAGAGTGCC
**5'end Ad4-VARNAI**	GTTCGCTTAGCCTCCAGGCCACGGAGTCGAGCC
**5'end Ad11-VARNAI**	CGTTCACGTTCCTCCAGGCTACGGAGTCGAGTC
**5'end Ad37- VARNAI**	TTTGCGTTCCGCCAGGCTACGGAGGAAGAGCCC
**tRNA-Lysine**	ACCGACTGAGCTATCCGGGC
**3'end Ad5-VARNAII**	AAAAAGGGGCTCGTCCCTGTTTCCGGAGG
**3'end Ad4-VARNAII**	AAAAGAGGGGCTCGCTCCGTAACTGGAGAAGTC
**3'end Ad37- VARNAII**	AAAAGGGGGCTCGCTCCCGTAACTGGAGGAGTC
**5'end Ad5-VARNAII**	AAATAACCCTCCGGCTACAGGGAGCGAGCC
**5'end Ad4-VARNAII**	TGGCGATTCTTCTCCAATCTACGGCAGTGAGCC
**5'end Ad37- VARNAII**	TGGCGATGCTTCTCCAGACTACGGGCGCGAGCC
