# Supplementary material for: Small RNA Sequence Analysis of Adenovirus VA RNA-Derived MiRNAs Reveals an Unexpected Serotype-Specific Difference in Structure and Abundance
Source: PLoS One. 2014 Aug 21;9(8):e105746. doi: 10.1371/journal.pone.0105746 (PMC4140831; doi:10.1371/journal.pone.0105746)
Supplement: Table S3 — List of total reads and length variations of the mivaRNAs expressed in the HAd infections. (PDF) [file pone.0105746.s008.pdf]

Supplementary Table 3. List of total reads and length variations of the mivaRNAs expressed in the HAd infections.

Ad5							
	Total mapped reads=84103				Total mapped reads=159089		
Cyto				RISC			
VA RNAI							
**Genomic Pos.**	**lenght**	**Read count**	**read count(%)**	**Genomic Pos.**	**lenght**	**Read count**	**read count(%)**
**-3**		**8444**	**10.04**	**-3**		**11864**	**7.46**
Ad_10617_10636	20	74	0.09	Ad_10617_10636	20	140	0.09
Ad_10617_10637	21	689	0.82	Ad_10617_10637	21	693	0.44
Ad_10617_10638	22	1178	1.40	Ad_10617_10638	22	1959	1.23
Ad_10617_10639	23	5887	7.00	Ad_10617_10639	23	8019	5.04
Ad_10617_10640	24	211	0.25	Ad_10617_10640	24	122	0.08
Ad_10617_10641	25	35	0.04	Ad_10617_10641	25	100	0.06
Ad_10617_10642	26	13	0.02	Ad_10617_10642	26	33	0.02
Ad_10617_10643	27	9	0.01	Ad_10617_10643	27	46	0.03
Ad_10617_10644	28	24	0.03	Ad_10617_10644	28	12	0.01
Ad_10617_10645	29	19	0.02	Ad_10617_10645	29	27	0.02
Ad_10617_10646	30	34	0.04	Ad_10617_10646	30	248	0.16
Ad_10617_10647	31	6	0.01	Ad_10617_10647	31	29	0.02
Ad_10617_10648	32	105	0.12	Ad_10617_10648	32	70	0.04
Ad_10617_10649	33	38	0.05	Ad_10617_10649	33	34	0.02
Ad_10617_10650	34	1	0.00	Ad_10617_10650	34	4	0.00
Ad_10617_10651	35	121	0.14	Ad_10617_10651	35	328	0.21
**-2**		**307**	**0.37**	**-2**		**493**	**0.31**
Ad_10618_10637	20	28	0.03	Ad_10618_10637	20	43	0.03
Ad_10618_10638	21	84	0.10	Ad_10618_10638	21	89	0.06
Ad_10618_10639	22	174	0.21	Ad_10618_10639	22	313	0.20
Ad_10618_10640	23	7	0.01	Ad_10618_10640	23	8	0.01
Ad_10618_10641	24	2	0.00	Ad_10618_10641	24	3	0.00
Ad_10618_10644	27	1	0.00	Ad_10618_10642	25	2	0.00
Ad_10618_10648	31	2	0.00	Ad_10618_10643	26	1	0.00
Ad_10618_10649	32	1	0.00	Ad_10618_10644	27	1	0.00
Ad_10618_10652	35	8	0.01	Ad_10618_10646	29	9	0.01
				Ad_10618_10647	30	1	0.00
				Ad_10618_10648	31	1	0.00
				Ad_10618_10649	32	5	0.00
				Ad_10618_10651	34	1	0.00
				Ad_10618_10652	35	16	0.01
**-1**		**105**	**0.12**	**-1**		**171**	**0.11**
Ad_10619_10638	20	7	0.01	Ad_10619_10638	20	25	0.02
Ad_10619_10639	21	54	0.06	Ad_10619_10639	21	68	0.04
Ad_10619_10640	22	36	0.04	Ad_10619_10640	22	50	0.03
Ad_10619_10641	23	3	0.00	Ad_10619_10641	23	17	0.01
Ad_10619_10643	25	1	0.00	Ad_10619_10646	28	2	0.00
Ad_10619_10653	35	4	0.00	Ad_10619_10649	31	1	0.00
				Ad_10619_10653	35	8	0.01
**1**		**7592**	**9.03**	**1**		**7925**	**4.98**
Ad_10620_10639	20	4248	5.05	Ad_10620_10639	20	1544	0.97
Ad_10620_10640	21	2640	3.14	Ad_10620_10640	21	1343	0.84
Ad_10620_10641	22	116	0.14	Ad_10620_10641	22	258	0.16
Ad_10620_10642	23	35	0.04	Ad_10620_10642	23	126	0.08
Ad_10620_10643	24	35	0.04	Ad_10620_10643	24	263	0.17
Ad_10620_10644	25	123	0.15	Ad_10620_10644	25	228	0.14
Ad_10620_10645	26	18	0.02	Ad_10620_10645	26	151	0.09
Ad_10620_10646	27	45	0.05	Ad_10620_10646	27	785	0.49
Ad_10620_10647	28	10	0.01	Ad_10620_10647	28	125	0.08
Ad_10620_10648	29	102	0.12	Ad_10620_10648	29	272	0.17
Ad_10620_10649	30	30	0.04	Ad_10620_10649	30	235	0.15
Ad_10620_10650	31	1	0.00	Ad_10620_10650	31	4	0.00
Ad_10620_10651	32	16	0.02	Ad_10620_10651	32	64	0.04
Ad_10620_10652	33	2	0.00	Ad_10620_10652	33	82	0.05
Ad_10620_10653	34	52	0.06	Ad_10620_10653	34	720	0.45
Ad_10620_10654	35	119	0.14	Ad_10620_10654	35	1725	1.08
**137**		**2455**	**2.92**	**137**		**9318**	**5.86**
Ad_10756_10775	20	110	0.13	Ad_10756_10775	20	562	0.35
Ad_10756_10776	21	493	0.59	Ad_10756_10776	21	1213	0.76
Ad_10756_10777	22	1149	1.37	Ad_10756_10777	22	4204	2.64
Ad_10756_10778	23	666	0.79	Ad_10756_10778	23	3207	2.02
Ad_10756_10779	24	36	0.04	Ad_10756_10779	24	130	0.08
Ad_10756_10782	27	1	0.00	Ad_10756_10780	25	2	0.00
**138**		**36807**	**43.76**	**138**		**78161**	**49.13**
Ad_10757_10776	20	1453	1.73	Ad_10757_10776	20	3154	1.98
Ad_10757_10777	21	8743	10.40	Ad_10757_10777	21	13799	8.67
Ad_10757_10778	22	25153	29.91	Ad_10757_10778	22	58377	36.69
Ad_10757_10779	23	1448	1.72	Ad_10757_10779	23	2777	1.75
Ad_10757_10780	24	7	0.01	Ad_10757_10780	24	40	0.03
Ad_10757_10784	28	3	0.00	Ad_10757_10781	25	6	0.00
				Ad_10757_10784	26	8	0.01
**139**		**2906**	**3.46**	**139**		**7105**	**4.47**
Ad_10758_10777	20	205	0.24	Ad_10758_10777	20	636	0.40
Ad_10758_10778	21	1987	2.36	Ad_10758_10778	21	5424	3.41
Ad_10758_10779	22	709	0.84	Ad_10758_10779	22	1038	0.65
Ad_10758_10780	23	4	0.00	Ad_10758_10780	23	5	0.00
Ad_10758_10784	27	1	0.00	Ad_10758_10781	24	1	0.00
				Ad_10758_10784	27	1	0.00
**140**		**5388**	**6.41**	**140**		**3879**	**2.44**
Ad_10759_10778	20	2381	2.83	Ad_10759_10778	20	1732	1.09
Ad_10759_10779	21	2976	3.54	Ad_10759_10779	21	2135	1.34
Ad_10759_10780	22	31	0.04	Ad_10759_10780	22	9	0.01
				Ad_10759_10782	24	1	0.00
				Ad_10759_10784	26	2	0.00

**Ad5**							
		Total mapped reads=84103				Total mapped reads=159089	
**Cyto**				**RISC**			
**VA RNAII**							
**Genomic Pos.**	**lenght**	**Read count**	**read count(%)**	**Genomic Pos.**	**lenght**	**Read count**	**read count(%)**
**1**		**6218**	**7.39**	**1**		**2988**	**1.88**
Ad_10876_10895	20	151	0.18	Ad_10876_10895	20	65	0.04
Ad_10876_10896	21	1177	1.40	Ad_10876_10896	21	2031	1.28
Ad_10876_10897	22	677	0.80	Ad_10876_10897	22	259	0.16
Ad_10876_10898	23	112	0.13	Ad_10876_10898	23	546	0.34
Ad_10876_10899	24	16	0.02	Ad_10876_10899	24	3	0.00
Ad_10876_10900	25	34	0.04	Ad_10876_10900	25	6	0.00
Ad_10876_10901	26	99	0.12	Ad_10876_10901	26	3	0.00
Ad_10876_10902	27	97	0.12	Ad_10876_10902	27	4	0.00
Ad_10876_10903	28	110	0.13	Ad_10876_10903	28	9	0.01
Ad_10876_10904	29	367	0.44	Ad_10876_10904	29	10	0.01
Ad_10876_10905	30	1311	1.56	Ad_10876_10905	30	44	0.03
Ad_10876_10906	31	193	0.23	Ad_10876_10907	32	5	0.00
Ad_10876_10907	32	1727	2.05	Ad_10876_10909	34	1	0.00
Ad_10876_10908	33	97	0.12	Ad_10876_10910	35	2	0.00
Ad_10876_10909	34	8	0.01				
Ad_10876_10910	35	42	0.05				
**136**		**979**	**1.16**	**136**		**24490**	**15.39**
Ad_11011_11030	20	23	0.03	Ad_11011_11030	20	266	0.17
Ad_11011_11031	21	146	0.17	Ad_11011_11031	21	4590	2.89
Ad_11011_11032	22	714	0.85	Ad_11011_11032	22	17562	11.04
Ad_11011_11033	23	79	0.09	Ad_11011_11033	23	1949	1.23
Ad_11011_11034	24	14	0.02	Ad_11011_11034	24	107	0.07
Ad_11011_11037	27	1	0.00	Ad_11011_11035	25	3	0.00
Ad_11011_11039	29	1	0.00	Ad_11011_11036	26	3	0.00
Ad_11011_11040	30	1	0.00	Ad_11011_11038	28	3	0.00
				Ad_11011_11039	29	2	0.00
				Ad_11011_11040	30	5	0.00
**138**		**3893**	**4.63**	**138**		**1551**	**0.97**
Ad_11012_11031	20	545	0.65	Ad_11012_11031	20	216	0.14
Ad_11012_11032	21	3057	3.63	Ad_11012_11032	21	986	0.62
Ad_11012_11033	22	277	0.33	Ad_11012_11033	22	335	0.21
Ad_11012_11034	23	4	0.00	Ad_11012_11034	23	9	0.01
Ad_11012_11035	24	6	0.01	Ad_11012_11035	24	1	0.00
Ad_11012_11036	25	1	0.00	Ad_11012_11036	25	1	0.00
Ad_11012_11037	26	1	0.00	Ad_11012_11037	26	1	0.00
Ad_11012_11038	27	2	0.00	Ad_11012_11038	27	1	0.00
				Ad_11012_11039	28	1	0.00
**139**		**2674**	**3.18**	**139**		**6470**	**4.07**
Ad_11013_11032	20	2006	2.39	Ad_11013_11032	20	1897	1.19
Ad_11013_11033	21	609	0.72	Ad_11013_11033	21	4117	2.59
Ad_11013_11034	22	46	0.05	Ad_11013_11034	22	388	0.24
Ad_11013_11035	23	6	0.01	Ad_11013_11035	23	63	0.04
Ad_11013_11036	24	6	0.01	Ad_11013_11036	24	4	0.00
Ad_11013_11037	25	1	0.00	Ad_11013_11037	25	1	0.00

Ad4

**Cyto**	Total Mapped reads: 24968			**RISC**		Total Mapped reads: 96629	
**VA RNAI**							
**Genomic Pos.**	**lenght**	**Read count**	**read count(%)**	**Genomic Pos.**	**lenght**	**Read count**	**read count(%)**
**-3**		**3021**	**12.15**	**-3**		**19447**	**20.13**
Ad_10354_10373	20	202	0.81	Ad_10354_10373	20	393	0.41
Ad_10354_10374	21	209	0.84	Ad_10354_10374	21	444	0.46
Ad_10354_10375	22	438	1.76	Ad_10354_10375	22	1873	1.94
Ad_10354_10376	23	1818	7.31	Ad_10354_10376	23	14912	15.43
Ad_10354_10377	24	133	0.53	Ad_10354_10377	24	1086	1.12
Ad_10354_10378	25	46	0.18	Ad_10354_10378	25	320	0.33
Ad_10354_10379	26	22	0.09	Ad_10354_10379	26	13	0.01
Ad_10354_10380	27	37	0.15	Ad_10354_10380	27	261	0.27
Ad_10354_10381	28	30	0.12	Ad_10354_10381	28	74	0.08
Ad_10354_10382	29	38	0.15	Ad_10354_10382	29	32	0.03
Ad_10354_10383	30	13	0.05	Ad_10354_10383	30	10	0.01
Ad_10354_10384	31	1	0.00	Ad_10354_10384	31	5	0.01
Ad_10354_10385	32	4	0.02	Ad_10354_10386	33	10	0.01
Ad_10354_10386	33	2	0.01	Ad_10354_10388	35	14	0.01
Ad_10354_10388	35	28	0.11				
**-2**		**25**	**0.10**	**-2**		**58**	**0.06**
Ad_10355_10374	20	1	0.00	Ad_10355_10374	20	2	0.00
Ad_10355_10375	21	3	0.01	Ad_10355_10375	21	2	0.00
Ad_10355_10376	22	12	0.05	Ad_10355_10376	22	42	0.04
Ad_10355_10377	23	4	0.02	Ad_10355_10377	24	9	0.01
Ad_10355_10378	24	1	0.00	Ad_10355_10378	26	1	0.00
Ad_10355_10380	26	3	0.01	Ad_10355_10379	27	2	0.00
Ad_10355_10383	29	1	0.00				0.12
**-1**		**15**	**0.06**	**-1**		**34**	**0.04**
Ad_10356_10375	20	1	0.00	Ad_10356_10375	20	3	0.00
Ad_10356_10376	21	8	0.03	Ad_10356_10376	21	13	0.01
Ad_10356_10377	22	2	0.01	Ad_10356_10377	22	13	0.01
Ad_10356_10380	25	1	0.00	Ad_10356_10381	26	3	0.00
Ad_10356_10382	27	1	0.00	Ad_10356_10387	32	1	0.00
Ad_10356_10385	30	1	0.00	Ad_10356_10390	35	1	0.00
Ad_10356_10386	31	1	0.00				
**1**		**590**	**2.37**	**1**		**773**	**0.80**
Ad_10357_10376	20	24	0.10	Ad_10357_10376	20	51	0.05
Ad_10357_10377	21	49	0.20	Ad_10357_10377	21	104	0.11
Ad_10357_10378	22	59	0.24	Ad_10357_10378	22	62	0.06
Ad_10357_10379	23	47	0.19	Ad_10357_10379	23	42	0.04
Ad_10357_10380	24	70	0.28	Ad_10357_10380	24	99	0.10
Ad_10357_10381	25	80	0.32	Ad_10357_10381	25	148	0.15
Ad_10357_10382	26	88	0.35	Ad_10357_10382	26	49	0.05
Ad_10357_10383	27	82	0.33	Ad_10357_10383	26	50	0.05
Ad_10357_10384	28	7	0.03	Ad_10357_10384	27	6	0.01
Ad_10357_10385	29	35	0.14	Ad_10357_10385	28	24	0.02
Ad_10357_10386	30	10	0.04	Ad_10357_10386	29	83	0.09
Ad_10357_10387	31	1	0.00	Ad_10357_10387	30	5	0.01
Ad_10357_10388	32	6	0.02	Ad_10357_10388	31	8	0.01
Ad_10357_10389	33	3	0.01	Ad_10357_10389	33	7	0.01
Ad_10357_10390	34	14	0.06	Ad_10357_10390	34	31	0.03
Ad_10357_10391	35	15	0.06	Ad_10357_10391	35	4	0.00
**137**		**112**	**0.45**	**137**		**1240**	**1.28**
Ad_10493_10512	20	9	0.04	Ad_10493_10512	20	143	0.15
Ad_10493_10513	21	3	0.01	Ad_10493_10513	21	40	0.04
Ad_10493_10514	22	26	0.10	Ad_10493_10514	22	435	0.45
Ad_10493_10515	23	59	0.24	Ad_10493_10515	23	607	0.63
Ad_10493_10516	24	9	0.04	Ad_10493_10516	24	13	0.01
Ad_10493_10517	25	5	0.02	Ad_10493_10517	25	2	0.00
Ad_10493_10518	26	1	0.00				
**138**				**138**		**219**	**0.23**
Ad_10494_10513	20	19	0.08	Ad_10494_10513	20	18	0.02
Ad_10494_10514	21	24	0.10	Ad_10494_10514	21	105	0.11
Ad_10494_10515	22	54	0.22	Ad_10494_10515	22	91	0.09
Ad_10494_10516	23	16	0.06	Ad_10494_10516	23	5	0.01
Ad_10494_10517	24	8	0.03				
Ad_10494_10518	25	3	0.01				
**139**		**1847**	**7.43**	**139**		**14704**	**15.22**
Ad_10495_10514	20	366	1.47	Ad_10495_10514	20	6408	6.63
Ad_10495_10515	21	620	2.49	Ad_10495_10515	21	2723	2.82
Ad_10495_10516	22	767	3.08	Ad_10495_10516	22	4744	4.91
Ad_10495_10517	23	84	0.34	Ad_10495_10517	23	780	0.81
Ad_10495_10518	24	10	0.04	Ad_10495_10518	24	46	0.05
				Ad_10495_10519	25	3	0.00
**140**		**1619**	**6.51**	**140**		**15282**	**15.82**
Ad_10496_10515	20	158	0.64	Ad_10496_10515	20	560	0.58
Ad_10496_10516	21	221	0.89	Ad_10496_10516	21	1586	1.64
Ad_10496_10517	22	460	1.85	Ad_10496_10517	22	6230	6.45
Ad_10496_10518	23	773	3.11	Ad_10496_10518	23	6856	7.10
Ad_10496_10519	24	5	0.02	Ad_10496_10519	24	15	0.02
Ad_10496_10520	25	1	0.00	Ad_10496_10520	25	34	0.04
Ad_10496_10523	28	1	0.00	Ad_10496_10529	34	1	0.00
**141**		**51**	**0.21**	**141**		**117**	**0.12**
Ad_10497_10516		8	0.03	Ad_10497_10516		22	0.02
Ad_10497_10517		23	0.09	Ad_10497_10517		45	0.05
Ad_10497_10518		19	0.08	Ad_10497_10518		50	0.05
Ad_10497_10520		1	0.00				
**142**		**66**	**0.27**	**142**		**400**	**0.41**
Ad_10498_10517		41	0.16	Ad_10498_10517		291	0.30
Ad_10498_10518		25	0.10	Ad_10498_10518		109	0.11

Ad4

Cyto Total Mapped reads: 24968

VA RNAII

**Genomic Pos.**	**lenght**	**Read count**	**read count(%)**
**-2**		**149**	**0.60**
Ad_10573_10592	20	5	0.02
Ad_10573_10593	21	1	0.00
Ad_10573_10594	22	2	0.01
Ad_10573_10595	23	21	0.08
Ad_10573_10596	24	24	0.10
Ad_10573_10597	25	86	0.34
Ad_10573_10601	28	2	0.01
Ad_10573_10605	32	1	0.00
Ad_10573_10606	33	4	0.02
Ad_10573_10607	34	3	0.01
**-1**		**33**	**0.13**
Ad_10574_10596	23	5	0.02
Ad_10574_10597	24	28	0.11
**1**		**1367**	**5.48**
Ad_10575_10594	20	8	0.03
Ad_10575_10595	21	7	0.03
Ad_10575_10596	22	203	0.81
Ad_10575_10597	23	1101	4.41
Ad_10575_10598	24	4	0.02
Ad_10575_10600	26	1	0.00
Ad_10575_10601	27	12	0.05
Ad_10575_10603	29	1	0.00
Ad_10575_10606	31	8	0.03
Ad_10575_10607	33	5	0.02
Ad_10575_10608	34	1	0.00
Ad_10575_10609	35	16	0.06
**147**		**931**	**3.73**
Ad_10721_10740	20	58	0.23
Ad_10721_10741	21	405	1.62
Ad_10721_10742	22	154	0.62
Ad_10721_10743	23	313	1.25
Ad_10721_10746	26	1	0.00
**148**		**31**	**0.12**
Ad_10722_10741	20	19	0.08
Ad_10722_10742	21	5	0.02
Ad_10722_10743	22	6	0.02
Ad_10722_10746	25	1	0.00
**149**			
Ad_10723_10744	22	2	0.01

RISC Total Mapped reads: 96629

**Genomic Pos.**	**lenght**	**Read count**	**read count(%)**
		**1557**	**1.61**
Ad_10573_10592	20	17	0.02
Ad_10573_10593	21	16	0.02
Ad_10573_10594	22	16	0.02
Ad_10573_10595	23	94	0.10
Ad_10573_10596	24	749	0.78
Ad_10573_10597	25	645	0.67
Ad_10573_10598	26	10	0.01
Ad_10573_10605	32	1	0.00
Ad_10573_10606	33	1	0.00
Ad_10573_10607	34	8	0.01
**-1**		**104**	**0.11**
Ad_10574_10595	22	6	0.01
Ad_10574_10596	23	19	0.02
Ad_10574_10597	24	79	0.08
**1**		**2892**	**2.99**
Ad_10575_10594	20	11	0.01
Ad_10575_10595	21	25	0.03
Ad_10575_10596	22	909	0.94
Ad_10575_10597	23	1930	2.00
Ad_10575_10598	24	6	0.01
Ad_10575_10601	27	3	0.00
Ad_10575_10602	28	3	0.00
Ad_10575_10609	35	5	0.01
**147**		**10865**	**11.24**
Ad_10721_10740	20	508	0.53
Ad_10721_10741	21	4824	4.99
Ad_10721_10742	22	858	0.89
Ad_10721_10743	23	4642	4.80
Ad_10721_10744	24	24	0.02
Ad_10721_10745	25	1	0.00
Ad_10721_10748	28	8	0.01
**148**		**574**	**0.59**
Ad_10722_10741	20	366	0.38
Ad_10722_10742	21	42	0.04
Ad_10722_10743	22	162	0.17
Ad_10722_10744	23	4	0.00
**149**		**20**	**0.02**
Ad_10723_10742	20	6	0.01
Ad_10723_10743	21	14	

Ad11

**Cyto Genomic Pos.**	Total mapped reads=842171 **lenght**	**Read count**	**read count(%)**
**-3**		**48699**	**5.78**
Ad_10429_10448	20	2880	0.34
Ad_10429_10449	21	5296	0.63
Ad_10429_10450	22	33294	3.95
Ad_10429_10451	23	4052	0.48
Ad_10429_10452	24	105	0.01
Ad_10429_10453	25	440	0.05
Ad_10429_10454	26	80	0.01
Ad_10429_10455	27	182	0.02
Ad_10429_10456	28	41	0.00
Ad_10429_10457	29	244	0.03
Ad_10429_10458	30	619	0.07
Ad_10429_10459	31	346	0.04
Ad_10429_10460	32	144	0.02
Ad_10429_10461	33	294	0.03
Ad_10429_10462	34	16	0.00
Ad_10429_10463	35	666	0.08
**-2**		**236**	**0.03**
Ad_10430_10449	20	18	0.00
Ad_10430_10450	21	84	0.01
Ad_10430_10451	22	54	0.01
Ad_10430_10452	23	1	0.00
Ad_10430_10453	24	13	0.00
Ad_10430_10454	25	8	0.00
Ad_10430_10456	27	1	0.00
Ad_10430_10457	28	10	0.00
Ad_10430_10458	29	14	0.00
Ad_10430_10459	30	5	0.00
Ad_10430_10460	31	4	0.00
Ad_10430_10461	32	10	0.00
Ad_10430_10463	34	2	0.00
Ad_10430_10464	35	12	0.00
**-1**		**747**	**0.09**
Ad_10431_10450	20	312	0.04
Ad_10431_10451	21	322	0.04
Ad_10431_10452	22	62	0.01
Ad_10431_10453	23	13	0.00
Ad_10431_10455	25	13	0.00
Ad_10431_10457	27	3	0.00
Ad_10431_10458	28	2	0.00
Ad_10431_10459	29	8	0.00
Ad_10431_10460	30	3	0.00
Ad_10431_10461	32	3	0.00
Ad_10431_10463	33	1	0.00
Ad_10431_10464	34	1	0.00
Ad_10431_10465	35	4	0.00
**1**		**79596**	**9.45**
Ad_10432_10451	20	6165	0.73
Ad_10432_10452	21	3042	0.36
Ad_10432_10453	22	8153	0.97
Ad_10432_10454	23	393	0.05
Ad_10432_10455	24	2025	0.24
Ad_10432_10456	25	207	0.02
Ad_10432_10457	26	2368	0.28
Ad_10432_10458	27	15265	1.81
Ad_10432_10459	28	19789	2.35
Ad_10432_10460	29	4355	0.52
Ad_10432_10461	30	4766	0.57
Ad_10432_10462	31	69	0.01
Ad_10432_10463	32	1161	0.14
Ad_10432_10464	33	3455	0.41
Ad_10432_10465	34	1823	0.22
Ad_10432_10466	35	6559	0.78
Ad_10432_10467	36	1	0.00
**138**		**384113**	**45.61**
Ad_10569_10588	20	62591	7.43
Ad_10569_10589	21	128873	15.30
Ad_10569_10590	22	177100	21.03
Ad_10569_10591	23	13459	1.60
Ad_10569_10592	24	2017	0.24
Ad_10569_10593	25	45	0.01
Ad_10569_10594	26	23	0.00
Ad_10569_10595	27	2	0.00
Ad_10569_10597	28	1	0.00
Ad_10569_10600	31	1	0.00
Ad_10569_10603	33	1	0.00
**139**		**51105**	**6.07**
Ad_10570_10589	20	11459	1.36
Ad_10570_10590	21	13280	1.58
Ad_10570_10591	22	15080	1.79
Ad_10570_10592	23	11149	1.32
Ad_10570_10593	24	61	0.01
Ad_10570_10594	25	74	0.01
Ad_10570_10595	26	1	0.00
Ad_10570_10596	27	1	0.00
**140**		**27145**	**3.22**
Ad_10571_10590	20	4955	0.59
Ad_10571_10591	21	17865	2.12
Ad_10571_10592	22	4237	0.50
Ad_10571_10593	23	23	0.00
Ad_10571_10594	24	63	0.01
Ad_10571_10595	25	1	0.00
Ad_10571_10597	27	1	0.00
**141**		**931**	**0.11**
Ad_10572_10591	20	721	0.09
Ad_10572_10592	21	207	0.02
Ad_10572_10595	24	3	0.00

**RISC Genomic Pos.**	Total mapped reads=2292737 **lenght**	**Read count**	**read count(%)**
**-3**		**183905**	**8.02**
Ad_10429_10448	20	8292	0.36
Ad_10429_10449	21	20951	0.91
Ad_10429_10450	22	86434	3.77
Ad_10429_10451	23	57778	2.52
Ad_10429_10452	24	4454	0.19
Ad_10429_10453	25	566	0.02
Ad_10429_10454	26	2113	0.09
Ad_10429_10455	27	782	0.03
Ad_10429_10456	28	165	0.01
Ad_10429_10457	29	699	0.03
Ad_10429_10458	30	1186	0.05
Ad_10429_10459	31	160	0.01
Ad_10429_10460	32	65	0.00
Ad_10429_10461	33	42	0.00
Ad_10429_10462	34	1	0.00
Ad_10429_10463	35	217	0.01
**-2**		**545**	**0.02**
Ad_10430_10449	20	44	0.00
Ad_10430_10450	21	138	0.01
Ad_10430_10451	22	222	0.01
Ad_10430_10452	23	12	0.00
Ad_10430_10453	24	12	0.00
Ad_10430_10454	25	46	0.00
Ad_10430_10455	26	22	0.00
Ad_10430_10456	27	6	0.00
Ad_10430_10457	28	8	0.00
Ad_10430_10458	29	25	0.00
Ad_10430_10459	30	2	0.00
Ad_10430_10461	32	3	0.00
Ad_10430_10463	34	2	0.00
Ad_10430_10464	35	3	0.00
**-1**		**12684**	**0.55**
Ad_10431_10450	20	1727	0.08
Ad_10431_10451	21	4867	0.21
Ad_10431_10452	22	5983	0.26
Ad_10431_10453	23	84	0.00
Ad_10431_10454	24	6	0.00
Ad_10431_10455	25	6	0.00
Ad_10431_10456	26	1	0.00
Ad_10431_10457	27	3	0.00
Ad_10431_10458	28	4	0.00
Ad_10431_10459	29	2	0.00
Ad_10431_10463	33	1	0.00
**1**		**74723**	**3.26**
Ad_10432_10451	20	17999	0.79
Ad_10432_10452	21	50062	2.18
Ad_10432_10453	22	566	0.02
Ad_10432_10454	23	568	0.02
Ad_10432_10455	24	404	0.02
Ad_10432_10456	25	59	0.00
Ad_10432_10457	26	172	0.01
Ad_10432_10458	27	849	0.04
Ad_10432_10459	28	1822	0.08
Ad_10432_10460	29	280	0.01
Ad_10432_10461	30	151	0.01
Ad_10432_10462	31	2	0.00
Ad_10432_10463	32	78	0.00
Ad_10432_10464	33	251	0.01
Ad_10432_10465	34	270	0.01
Ad_10432_10466	35	1190	0.05
**138**		**1537308**	**67.05**
Ad_10569_10588	20	135595	5.91
Ad_10569_10589	21	360627	15.73
Ad_10569_10590	22	999936	43.61
Ad_10569_10591	23	39606	1.73
Ad_10569_10592	24	1429	0.06
Ad_10569_10593	25	77	0.00
Ad_10569_10594	26	33	0.00
Ad_10569_10595	27	1	0.00
Ad_10569_10596	28	1	0.00
Ad_10569_10597	29	1	0.00
Ad_10569_10598	30	1	0.00
Ad_10569_10599	31	1	0.00
**139**		**314781**	**13.73**
Ad_10570_10589	20	20434	0.89
Ad_10570_10590	21	78522	3.42
Ad_10570_10591	22	120733	5.27
Ad_10570_10592	23	94476	4.12
Ad_10570_10593	24	286	0.01
Ad_10570_10594	25	316	0.01
Ad_10570_10595	26	5	0.00
Ad_10570_10596	27	3	0.00
Ad_10570_10597	28	4	0.00
Ad_10570_10598	29	2	0.00
**140**		**56647**	**2.47**
Ad_10571_10590	20	5883	0.26
Ad_10571_10591	21	40662	1.77
Ad_10571_10592	22	9934	0.43
Ad_10571_10593	23	34	0.00
Ad_10571_10594	24	133	0.01
Ad_10571_10595	25	1	0.00
**142**		**12107**	**0.53**
Ad_10572_10591	20	8731	0.38
Ad_10572_10592	21	3352	0.15
Ad_10572_10593	22	8	0.00
Ad_10572_10594	24	16	0.00

Ad37

**Cyto**	Total mapped reads=345919		
**VA RNAI**			
**Genomic Pos.**	**lenght**	**Read count**	**read count(%)**
**1**		**5704**	**1.65**
Ad_10252_10271	20	153	0.04
Ad_10252_10272	21	666	0.19
Ad_10252_10273	22	530	0.15
Ad_10252_10274	23	672	0.19
Ad_10252_10275	24	1714	0.50
Ad_10252_10276	25	17	0.00
Ad_10252_10277	26	26	0.01
Ad_10252_10278	27	12	0.00
Ad_10252_10279	28	41	0.01
Ad_10252_10280	29	21	0.01
Ad_10252_10281	30	224	0.06
Ad_10252_10282	31	407	0.12
Ad_10252_10283	32	326	0.09
Ad_10252_10284	33	29	0.01
Ad_10252_10285	34	63	0.02
Ad_10252_10286	35	803	0.23
**139**		**691**	**0.20**
Ad_10390_10409	20	94	0.03
Ad_10390_10410	21	184	0.05
Ad_10390_10411	22	167	0.05
Ad_10390_10412	23	146	0.04
Ad_10390_10413	24	83	0.02
Ad_10390_10414	25	11	0.00
Ad_10390_10415	26	6	0.00
**140**		**54804**	**15.84**
Ad_10391_10410	20	1488	0.43
Ad_10391_10411	21	27568	7.97
Ad_10391_10412	22	15160	4.38
Ad_10391_10413	23	9752	2.82
Ad_10391_10414	24	756	0.22
Ad_10391_10415	25	75	0.02
Ad_10391_10416	26	5	0.00
**141**		**32993**	**9.54**
Ad_10392_10411	20	11730	3.39
Ad_10392_10412	21	12673	3.66
Ad_10392_10413	22	8067	2.33
Ad_10392_10414	23	478	0.14
Ad_10392_10415	24	41	0.01
Ad_10392_10416	25	3	0.00
Ad_10392_10417	26	1	0.00
**142**		**206**	**0.06**
Ad_10393_10412	20	93	0.03
Ad_10393_10413	21	94	0.03
Ad_10393_10414	22	15	0.00
Ad_10393_10415	23	4	0.00

**RISC**	Total mapped reads=867611		
**Genomic Pos.**	**lenght**	**Read count**	**read count(%)**
**1**		**32020**	**3.69**
Ad_10252_10271	20	951	0.11
Ad_10252_10272	21	1820	0.21
Ad_10252_10273	22	2473	0.29
Ad_10252_10274	23	1780	0.21
Ad_10252_10275	24	8415	0.97
Ad_10252_10276	25	215	0.02
Ad_10252_10277	26	231	0.03
Ad_10252_10278	27	84	0.01
Ad_10252_10279	28	575	0.07
Ad_10252_10280	29	632	0.07
Ad_10252_10281	30	2597	0.30
Ad_10252_10282	31	5601	0.65
Ad_10252_10283	32	1743	0.20
Ad_10252_10284	33	86	0.01
Ad_10252_10285	34	608	0.07
Ad_10252_10286	35	4209	0.49
**139**		**3005**	**0.35**
Ad_10390_10409	20	574	0.07
Ad_10390_10410	21	1446	0.17
Ad_10390_10411	22	392	0.05
Ad_10390_10412	23	236	0.03
Ad_10390_10413	24	297	0.03
Ad_10390_10414	25	40	0.00
Ad_10390_10415	26	20	0.00
**140**		**61070**	**7.04**
Ad_10391_10410	20	8856	1.02
Ad_10391_10411	21	14770	1.70
Ad_10391_10412	22	15876	1.83
Ad_10391_10413	23	20371	2.35
Ad_10391_10414	24	1052	0.12
Ad_10391_10415	25	141	0.02
Ad_10391_10416	26	4	0.00
**141**		**55815**	**6.43**
Ad_10392_10411	20	16609	1.91
Ad_10392_10412	21	14427	1.66
Ad_10392_10413	22	23511	2.71
Ad_10392_10414	23	1118	0.13
Ad_10392_10415	24	121	0.01
Ad_10392_10416	25	24	0.00
Ad_10392_10417	26	2	0.00
Ad_10392_10419	28	3	0.00
**142**		**143**	**0.02**
Ad_10393_10412	20	64	0.01
Ad_10393_10413	21	77	0.01
Ad_10393_10414	22	1	0.00
Ad_10393_10415	23	1	0.00

Ad37

	Total mapped				Total mapped		
**Cyto**	reads=345919			**RISC**	reads=867611		
**VA RNAII**							
**Genomic Pos.**	**lenght**	**Read count**	**read count(%)**	**Genomic Pos.**	**lenght**	**Read count**	**read count(%)**
**-3**		**6301**	**1.82**	**-3**		**194154**	**22.38**
Ad_10468_10487	20	508	0.15	Ad_10468_10487	20	8653	1.00
Ad_10468_10488	21	564	0.16	Ad_10468_10488	21	4103	0.47
Ad_10468_10489	22	1207	0.35	Ad_10468_10489	22	55635	6.41
Ad_10468_10490	23	1057	0.31	Ad_10468_10490	23	34211	3.94
Ad_10468_10491	24	2511	0.73	Ad_10468_10491	24	75185	8.67
Ad_10468_10492	25	213	0.06	Ad_10468_10492	25	14066	1.62
Ad_10468_10493	26	66	0.02	Ad_10468_10493	26	2156	0.25
Ad_10468_10494	27	4	0.00	Ad_10468_10494	27	56	0.01
Ad_10468_10495	28	2	0.00	Ad_10468_10495	28	8	0.00
Ad_10468_10496	29	13	0.00	Ad_10468_10496	29	10	0.00
Ad_10468_10497	30	49	0.01	Ad_10468_10497	30	43	0.00
Ad_10468_10498	31	2	0.00	Ad_10468_10499	32	1	0.00
Ad_10468_10500	33	2	0.00	Ad_10468_10502	35	27	0.00
Ad_10468_10501	34	4	0.00				
Ad_10468_10502	35	99	0.03				
**-2**		**466**	**0.13**	**-2**		**19599**	**2.26**
Ad_10469_10488	20	23	0.01	Ad_10469_10488	20	325	0.04
Ad_10469_10489	21	45	0.01	Ad_10469_10489	21	4461	0.51
Ad_10469_10490	22	32	0.01	Ad_10469_10490	22	1591	0.18
Ad_10469_10491	23	209	0.06	Ad_10469_10491	23	9110	1.05
Ad_10469_10492	24	107	0.03	Ad_10469_10492	24	3921	0.45
Ad_10469_10493	25	19	0.01	Ad_10469_10493	25	188	0.02
Ad_10469_10496	28	6	0.00	Ad_10469_10494	26	3	0.00
Ad_10469_10497	29	5	0.00				
Ad_10469_10501	30	3	0.00				
Ad_10469_10502	31	5	0.00				
Ad_10469_10503	32	12	0.00				
**-1**		**41**	**0.01**	**-1**		**600**	**0.07**
Ad_10470_10489	20	3	0.00	Ad_10470_10489	20	42	0.00
Ad_10470_10490	21	1	0.00	Ad_10470_10490	21	19	0.00
Ad_10470_10491	22	5	0.00	Ad_10470_10491	22	98	0.01
Ad_10470_10492	23	21	0.01	Ad_10470_10492	23	318	0.04
Ad_10470_10493	24	7	0.00	Ad_10470_10493	24	123	0.01
Ad_10470_10497	28	1	0.00				
Ad_10470_10501	32	3	0.00				
**1**		**7377**	**2.13**	**1**		**116013**	**13.37**
Ad_10471_10490	20	49	0.01	Ad_10471_10490	20	2234	0.26
Ad_10471_10491	21	416	0.12	Ad_10471_10491	21	9474	1.09
Ad_10471_10492	22	4993	1.44	Ad_10471_10492	22	89493	10.31
Ad_10471_10493	23	1586	0.46	Ad_10471_10493	23	14187	1.64
Ad_10471_10494	24	11	0.00	Ad_10471_10494	24	307	0.04
Ad_10471_10495	25	2	0.00	Ad_10471_10495	25	2	0.00
Ad_10471_10496	26	10	0.00	Ad_10471_10496	26	79	0.01
Ad_10471_10497	27	69	0.02	Ad_10471_10497	27	76	0.01
Ad_10471_10498	28	1	0.00	Ad_10471_10499	29	52	0.01
Ad_10471_10499	29	8	0.00	Ad_10471_10500	30	4	0.00
Ad_10471_10500	30	3	0.00	Ad_10471_10502	32	68	0.01
Ad_10471_10501	31	1	0.00	Ad_10471_10504	34	3	0.00
Ad_10471_10502	32	32	0.01	Ad_10471_10505	35	34	0.00
Ad_10471_10503	33	1	0.00				
Ad_10471_10504	34	6	0.00				
Ad_10471_10505	35	189	0.05				

**128**		**10035**	**2.90**
Ad_10598_10617	20	360	0.10
Ad_10598_10618	21	2418	0.70
Ad_10598_10619	22	4696	1.36
Ad_10598_10620	23	1645	0.48
Ad_10598_10621	24	463	0.13
Ad_10598_10622	25	391	0.11
Ad_10598_10623	26	19	0.01
Ad_10598_10624	27	29	0.01
Ad_10598_10625	28	11	0.00
Ad_10598_10626	29	2	0.00
Ad_10598_10627	30	1	0.00
**129**		**10357**	**2.99**
Ad_10599_10618	20	1665	0.48
Ad_10599_10619	21	4820	1.39
Ad_10599_10620	22	3380	0.98
Ad_10599_10621	23	255	0.07
Ad_10599_10622	24	185	0.05
Ad_10599_10623	25	16	0.00
Ad_10599_10624	26	17	0.00
Ad_10599_10625	27	14	0.00
Ad_10599_10626	28	2	0.00
Ad_10599_10627	29	3	0.00
**130**		**14238**	**4.12**
Ad_10600_10619	20	3868	1.12
Ad_10600_10620	21	7374	2.13
Ad_10600_10621	22	2589	0.75
Ad_10600_10622	23	264	0.08
Ad_10600_10623	24	65	0.02
Ad_10600_10624	25	57	0.02
Ad_10600_10625	26	12	0.00
Ad_10600_10626	27	2	0.00
Ad_10600_10627	28	7	0.00
**131**		**802**	**0.23**
Ad_10601_10620	20	180	0.05
Ad_10601_10621	21	212	0.06
Ad_10601_10622	22	366	0.11
Ad_10601_10623	23	29	0.01
Ad_10601_10624	24	9	0.00
Ad_10601_10625	25	6	0.00

**128**		**153392**	**17.68**
Ad_10598_10617	20	15661	1.81
Ad_10598_10618	21	44391	5.12
Ad_10598_10619	22	65145	7.51
Ad_10598_10620	23	10351	1.19
Ad_10598_10621	24	7817	0.90
Ad_10598_10622	25	9136	1.05
Ad_10598_10623	26	267	0.03
Ad_10598_10624	27	397	0.05
Ad_10598_10625	28	162	0.02
Ad_10598_10626	29	30	0.00
Ad_10598_10627	30	19	0.00
Ad_10598_10628	31	12	0.00
Ad_10598_10629	32	2	0.00
Ad_10598_10632	35	2	0.00
**129**		**42892**	**4.94**
Ad_10599_10618	20	11033	1.27
Ad_10599_10619	21	21426	2.47
Ad_10599_10620	22	4977	0.57
Ad_10599_10621	23	2421	0.28
Ad_10599_10622	24	2897	0.33
Ad_10599_10623	25	50	0.01
Ad_10599_10624	26	37	0.00
Ad_10599_10625	27	33	0.00
Ad_10599_10626	28	12	0.00
Ad_10599_10627	29	5	0.00
Ad_10599_10630	32	1	0.00
**130**		**15074**	**1.74**
Ad_10600_10619	20	1385	0.16
Ad_10600_10620	21	5951	0.69
Ad_10600_10621	22	6511	0.75
Ad_10600_10622	23	779	0.09
Ad_10600_10623	24	274	0.03
Ad_10600_10624	25	105	0.01
Ad_10600_10625	26	33	0.00
Ad_10600_10626	27	1	0.00
Ad_10600_10627	28	19	0.00
Ad_10600_10628	29	16	0.00
**131**		**5583**	**0.64**
Ad_10601_10620	20	807	0.09
Ad_10601_10621	21	1931	0.22
Ad_10601_10622	22	2243	0.26
Ad_10601_10623	23	343	0.04
Ad_10601_10624	24	217	0.03
Ad_10601_10625	25	14	0.00
Ad_10601_10627	27	13	0.00
Ad_10601_10628	28	13	0.00
Ad_10601_10630	30	1	0.00
Ad_10601_10632	32	1	0.00
